# Mouse screen reveals multiple new genes underlying mouse and human hearing loss

**DOI:** 10.1371/journal.pbio.3000194

**Published:** 2019-04-11

**Authors:** Neil J. Ingham, Selina A. Pearson, Valerie E. Vancollie, Victoria Rook, Morag A. Lewis, Jing Chen, Annalisa Buniello, Elisa Martelletti, Lorenzo Preite, Chi Chung Lam, Felix D. Weiss, Zӧe Powis, Pim Suwannarat, Christopher J. Lelliott, Sally J. Dawson, Jacqueline K. White, Karen P. Steel

**Affiliations:** 1 Wellcome Trust Sanger Institute, Hinxton, United Kingdom; 2 Wolfson Centre for Age-Related Diseases, Institute of Psychiatry, Psychology and Neuroscience, King’s College London, London, United Kingdom; 3 Department of Emerging Genetics Medicine, Ambry Genetics, Aliso Viejo, California, United States of America; 4 Mid-Atlantic Permanente Medical Group, Rockville, Maryland, United States of America; 5 UCL Ear Institute, University College London, London, United Kingdom; University of Edinburgh, UNITED KINGDOM

## Abstract

Adult-onset hearing loss is very common, but we know little about the underlying molecular pathogenesis impeding the development of therapies. We took a genetic approach to identify new molecules involved in hearing loss by screening a large cohort of newly generated mouse mutants using a sensitive electrophysiological test, the auditory brainstem response (ABR). We review here the findings from this screen. Thirty-eight unexpected genes associated with raised thresholds were detected from our unbiased sample of 1,211 genes tested, suggesting extreme genetic heterogeneity. A wide range of auditory pathophysiologies was found, and some mutant lines showed normal development followed by deterioration of responses, revealing new molecular pathways involved in progressive hearing loss. Several of the genes were associated with the range of hearing thresholds in the human population and one, *SPNS2*, was involved in childhood deafness. The new pathways required for maintenance of hearing discovered by this screen present new therapeutic opportunities.

## Abstract 2

This study uses an electrophysiological screen of over a thousand new mutant mouse lines to identify 38 new genes underlying deafness, some associated with human hearing function, revealing a wide range of molecular and pathological mechanisms.

## Author summary

Progressive hearing loss with age is extremely common in the population, leading to difficulties in understanding speech, increased social isolation, and associated depression. We know it has a significant heritability, but so far we know very little about the molecular pathways leading to hearing loss, hampering the development of treatments. Here, we describe a large-scale screen of 1,211 new targeted mouse mutant lines, resulting in the identification of 38 genes underlying hearing loss that were not previously suspected of involvement in hearing. Some of these genes reveal molecular pathways that may be useful targets for drug development. Our further analysis of the genes identified and the varied pathological mechanisms within the ear resulting from the mutations suggests that hearing loss is an extremely heterogeneous disorder and may have as many as 1,000 genes involved.

## Introduction

Hearing loss is a very common disorder with a significant social impact, including delayed speech and language development, reduced academic achievement, increased social isolation, and risk of depression, and has recently been reported to be a major risk factor for dementia [1], adding new impetus to the need to develop therapies. Approximately 1 in 850 children are born with permanent hearing impairment in the United Kingdom [2], and the number of people affected by adult-onset hearing loss increases with each decade of life, with 60% of people in their 70s having a hearing loss of 25 dB or worse [3]. Environmental factors including noise or drug exposure play an important role in its etiology, but there is also a strong genetic contribution. Over 360 genes are known to be involved in human or mouse deafness, but ascertainment bias has led to many of these having early developmental effects, and little is known about the genetic contribution to adult-onset hearing loss. We set out to identify further genes underlying deafness, including those with mild effects, using a physiological screen based on the auditory brainstem response (ABR) in a large cohort of newly generated targeted mouse mutants. In this report, we review the findings from this screen and present the new data; both mice and ABR waveform data are available for further analysis. From the unbiased sample of 1,211 genes tested, we found 38 unexpected genes to be involved in hearing impairment. This indicates that around 600 additional genes remain to be found (see later), making deafness an extremely heterogeneous condition, with around 1,000 genes that may contribute. The observed impairments ranged from mild to profound, including several with progressive hearing loss, and with a wide range of underlying pathological mechanisms. The 38 genes represent a range of functions from transcription factors and a microRNA to enzymes involved in lipid metabolism. Eleven were found to be significantly associated with auditory function in the human population, and one gene, *SPNS2*, was associated with childhood deafness, emphasising the value of the mouse for identifying genes and mechanisms underlying complex processes like hearing.

## Results and discussion

### Genes involved in auditory sensitivity

**Fig 1:**
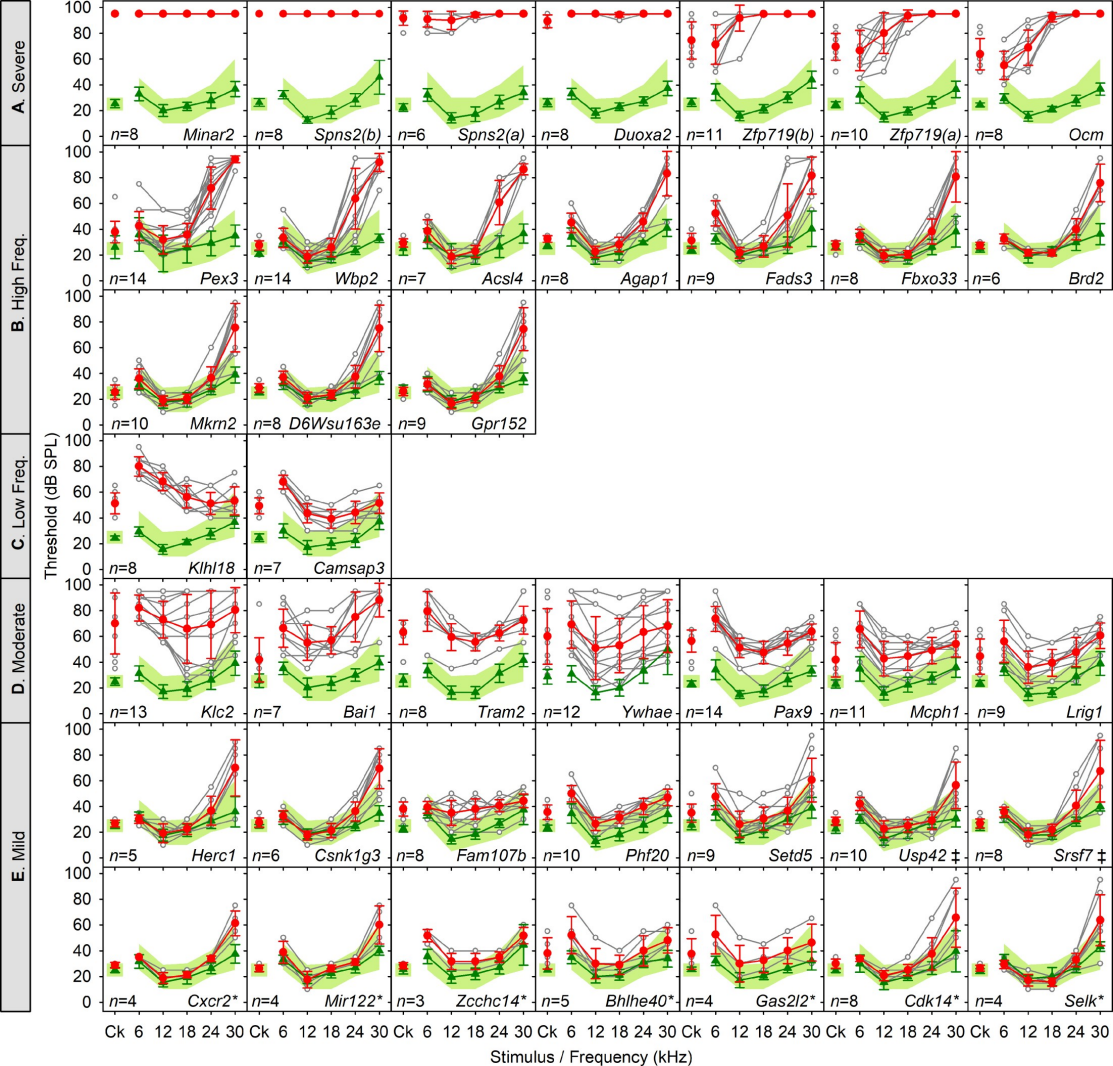
Novel genes affecting hearing sensitivity. New mutant mouse lines with hearing impairment categorised as Severe–Profound (A), High frequency (B), Low frequency (C), Moderate (D), and Mild (E) at 14 weeks old. Mean ABR thresholds (±SD) are plotted for broadband clicks (Ck) and 6 to 30 kHz pure tone stimuli. On each panel, the green band denotes the 95% reference range for a large population of control wild-type mice derived from littermates of the mutants generated and tested. Green lines and triangles represent the mean thresholds (±SD) for control mice recorded in the same week as the mutants. Red circles and lines represent the mean thresholds (±SD) for mutant mice. Thresholds for individual mutants are shown by open grey circles and lines. Gene symbols are given on each plot, and when both the *tm1a* and *tm1b* alleles were screened, both sets of data are presented, indicated by (a) and (b) suffixes. Mice screened were homozygous mutants except for *Brd2*, *Srsf7*, and *Setd5*, which were screened as heterozygotes due to reduced viability of homozygotes. When no response was detected up to the maximum dB SPL used (95 dB SPL), the maximum sound level used was plotted. Only 12 of these mutant lines (35%) would have been identified as having a hearing defect had startle responses alone been used to screen (S1 Table). The number of mutant mice screened of each line is given in column E of S1 Table and on each panel. A few of these lines (*Spns2*, *Wbp2*, *Lrig1*, *Ocm*, *Mcph1*, *Pax9*, or *Slc25a21*) have been characterised and published, and others were listed in a summary report (*Zcchc14*, *Adgrb1*, *Tram2*, *Klc2*, *Acsl4*, *Gpr152*, *Klhl18*, *Zfp719*, *A730017C20Rik/Minar2*, and *Duoxa2*) [7] because they were first detected in the current screen, but are included in the group analysis here, as they were not known to be involved in auditory function before the screen. Asterisks indicate lines that were called using the reference range or 20 dB rules described in the Materials and methods but were not significant calls using the Fisher Exact test; ‡ indicates lines that were not significant by the Fisher Exact test but did show raised thresholds when other cohorts were tested later. Plotted data points are given S5 Data. ABR, auditory brainstem response; Ck, click; SPL, sound pressure level.

We used a rapid (15-minute), noninvasive electrophysiological test, the ABR [4] (S1 Fig; S1 Data) in anaesthetised mice aged 14 weeks old as part of an extensive pipeline of phenotyping tests on a set of new mouse mutants generated from targeted embryonic stem (ES) cells [5,6,7]. The allele design was mostly the knockout first, conditional-ready (*tm1a*; targeted mutation, first allele with design type a [6]) allele, which reduced or eliminated expression of the targeted gene by inclusion of a large cassette designed to interfere with transcription, but a few were the derived *tm1b* allele with an exon deleted or were edited alleles (S1 and S2 Tables). A total of 1,211 genes were tested. Of these, 38 genes with no prior association with deafness had raised thresholds for detecting a response to sounds (Fig 1; a small number of these have been published recently, after their discovery in the screen). Using objective criteria (see Materials and methods) we classified these 38 genes into five main groups based on thresholds: 5 showed severe or profound deafness, 10 had raised thresholds at high frequencies only, 2 showed raised thresholds at low frequencies only, 7 had moderately raised thresholds across frequencies, and 14 had a mild hearing impairment (Fig 1). In addition to these 38 unexpected genes, 10 known deaf mutant lines were tested as positive controls (S2 Fig A-J; S2 Data), and 9 genes known to be involved in mouse (*Srrm4*) or human deafness (*MYO7A*, *MYO15*, *USH1C*, *WHRN*, *ILDR1*, *ESPN*, *CEP250*, *CLPP*) were tested in newly generated alleles; all showed raised thresholds (S1 Table; S2K–S2T Fig; S2 Data).

Of the set of 1,211 genes, 3.14% were new associations with raised auditory thresholds. As the genes targeted were an unbiased set showing no significant enrichment for any functional class compared with the total set of mouse genes (see Materials and methods), we can extrapolate to estimate that over 600 further genes required for normal auditory thresholds remain to be found. Added to the 362 human and mouse genes already known and 38 reported here, this indicates that there may be as many as 1,000 genes involved in deafness, a very high level of genetic heterogeneity.

There were several targeted genes screened that we expected to show raised thresholds because they had previously been reported to underlie deafness in either humans (*GSDME/DFNA5*, *MYH14*, *MYH9*, *PNPT1*, *PRPS1*, *CHD7)* or mice (*Barhl1*, *Fzd6*, *Hmx3*, *Nfkb1*, *Sgms1*, *Sms*, *Synj2)*. However, the new alleles had normal ABR thresholds (S3 Table). The lack of raised thresholds could be due to incomplete knockdown of targeted gene expression in the *tm1a* allele (e.g., raised thresholds of *Selk* mutants were seen only in the *tm1b* allele, not in *tm1a*); onset of hearing loss after 14 weeks, when we carried out the screening; screening was carried out on heterozygotes due to reduced homozygote viability; the genetic background may have influenced phenotype expression (e.g., *Chd7*, where the new allele was not viable on the C57BL/6N background); or the original deafness may have been the result of a specific effect of the mutation on the protein rather than a consequence of reduced expression (e.g., *GSDME*) (S3 Table). Alternatively, the original allele might have led to deafness via a long-range *cis* effect on a nearby gene, as in the *Slc25a21*^*tm1a(KOMP)Wtsi*^ targeted mutation, which causes deafness by reducing expression of *Pax9* [8]. Thus, we probably missed additional genes involved in hearing loss, so our calculation of 600 more genes awaiting association with deafness may be an underestimate.

All mutant mice reported here are available via public mouse repositories for further investigation to explore these alternative explanations, and the unprocessed ABR data are available at the Dryad repository: http://dx.doi.org/10.5061/dryad.cv803rv [9]. Of note, all of the mutant lines that we have studied further following the initial screening results have shown raised ABR thresholds, even those in the mild class, suggesting that the screen has produced robust, reproducible calls (mutant lines studied further: *Spns2*, *Zfp719*, *Ocm*, *Klhl18*, *Wbp2*, *Pex3*, *Acsl4*, *Gpr152*, *Mcph1*, *Slc25a21/Pax9*, *Ywhae*, *Lrig1*, *Klc2*, *Usp42*, *Srsf7)* [8, 10, 11, 12].

### Role in human auditory thresholds

As mouse and human inner ears are very similar in structure and function (e.g., [13]), the newly identified mouse genes represent good candidates for involvement in human deafness. A child from a United States clinic detected through clinical whole exome sequencing inherited a frameshift mutation of *SPNS2* from her father (c.1066_1067delCCinsT: p.Pro356Cysfs*35; CADD phred score 26) and an in-frame deletion of a serine codon in *SPNS2* from her mother (c.955_957delTCC: p.Ser319del; CADD phred score 20.9). Neither variant has been reported in the gnomAD database (Genome Aggregation Database; accessed January 2019). Visual reinforcement audiometry at two years old revealed moderate to moderately severe hearing loss between 250 Hz and 4 kHz with no response at 8 kHz in the right ear, and severe hearing loss sloping to profound deafness from 500 Hz to 8 kHz with no response at 4 and 8 kHz in the left ear. Bone conduction testing indicated moderate-severe hearing loss at 2 kHz in the right ear, suggesting a sensorineural (not conductive) impairment, and acoustic reflexes were absent. However, the child had surprisingly good sound localisation performance. The severe level of hearing impairment associated with predicted damaging *SPNS2* variants is similar to our findings in the mouse *Spns2* mutant (Fig 1 in [11]). Furthermore, we have previously reported that deaf children from two families in a Chinese cohort carried recessive *WBP2* mutations [10].

We asked if these new candidates had any role in hearing ability in the general population by a candidate gene association analysis. We tested genomic markers within 0.1 Mb up- and downstream of each gene for association with auditory thresholds measured at age 44–45 in 6,099 individuals born during one week in the UK 1958 British Birth Cohort, using genetic data imputed to the 1,000 Genomes dataset [14]. Eleven of the thirty-seven candidate genes tested (including *SPNS2*) showed a significant association of markers with threshold at either 1 or 4 kHz or both frequencies (Table 1), indicating that these 11 genes may play a role in normal variation of hearing ability in the human population.

**Table 1:** Significant associations of variants close to candidate genes with 1 kHz or 4 kHz auditory thresholds in the UK 1958 Birth Cohort. The *p*-values above the significance threshold of *p* ≤ 6.76 × 10^−4^ are not shown, and 37 of the 38 candidate genes were tested; no human orthologue is known for *Mir122*, so this was not included.

	Lowest *p*-value within gene			Lowest *p*-value within 0.1 Mb of gene		
	*p*-value	Trait	Variant	*p*-value	Trait	Variant
***AGAP1***	0.000213	4 kHz	rs34690321			
** **	0.0000366	1 kHz	2:236471205:TTTC_T			
***BAI1***				0.000304	4 kHz	rs34492225
** **				0.000309	1 kHz	rs77210320
***CDK14***				0.0000818	4 kHz	rs185413613
***LRIG1***	0.000628	1 kHz	rs140605629			
***MCPH1***	0.000277	4 kHz	rs2442589			
***MKRN2***				0.0000624	4 kHz	rs11718084
** **				0.000309	1 kHz	rs9834598
***PEX3***	0.000186	4 kHz	rs161072	0.000166	4 kHz	rs223228
***PHF20***	0.000309	1 kHz	rs187134453			
***SETD5***				0.000235	1 kHz	rs138835191
				0.000516	4 kHz	rs2728943
***SPNS2***				0.000204	4 kHz	rs117002379
***TRAM2***	0.000438	4 kHz	rs32073184	0.0000768	4 kHz	rs6458844

These findings emphasise the value of the mouse in resolving complex human diseases, because very few significant markers have been reported to be linked to hearing through genome-wide association studies of adult-onset hearing loss directly in humans: *GRM7* [15]; *PCDH20* and *SLC28A3* [16]; *ISG20* or *ACAN* and *TRIOBP* [17].

Several of the new genes that we found to be involved in deafness in the mouse had human orthologues close to or within unidentified non-syndromic deafness loci (S4 Table, column J) and so are good candidates for further exploration. These were *USP42* within the *DFNB90* interval, *BRD2* close to the *DFNA21* and *DFNA31* loci, *CAMSAP3* close to the *DFNA57* region, and *MCPH1* very close to the *DFNM2* marker reported.

### Broad range of gene functions

The 38 genes newly associated with hearing are involved in a broad range of functions, including transcriptional and translational regulation, chromatin modification, splicing factors, cytoskeletal proteins, membrane trafficking, calcium buffering, peroxisome biosynthesis, thyroid hormone generation, ubiquitination and deubiquitination, kinases, signaling molecules (including Wnt signaling), and proteins with no known or predicted function (S4 Table). A microRNA gene, *Mir122*, was one of the new genes underlying hearing impairment. Seven of the gene products have a role in lipid metabolism: Fads3 is a fatty acid desaturase; Agap1 and Zcchc14 bind phospholipids; Klc2 transports phosphatidylinositol 3-kinase, which is required for phospholipid processing; Pex3 is involved in biosynthesis of peroxisomes, which are involved in lipid processing; Acsl4 is a long-chain fatty acid coenzyme A ligase converting free long-chain fatty acids into fatty acyl-CoA esters; and Spns2 is a transporter of sphingosine-1-phosphate, a key intermediate in sphingolipid metabolism with a role in signalling.

**Fig 2:**
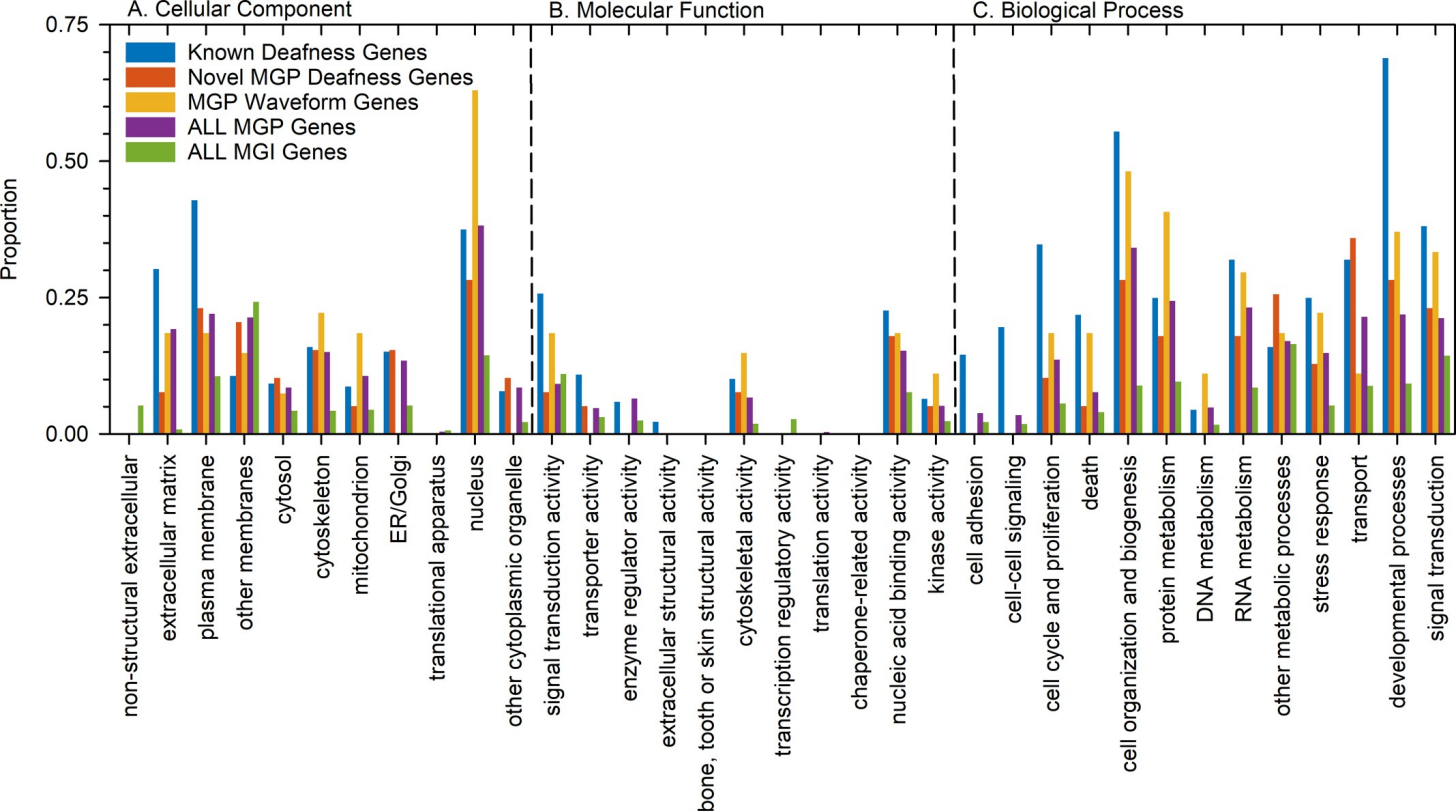
GOSlim comparisons of gene groups. GO terms associated with each gene were compared between the five groups: genes previously known to be associated with deafness in human and mouse (*n* = 362); novel genes associated with raised ABR thresholds in the current screen (*n* = 38); genes with normal thresholds but abnormal waveform features (*n* = 27); all genes screened in the MGP screen (*n* = 1,211); and all genes listed in MGI (*n* = 33,395). The proportion of genes in each group with the high-level GO terms listed are plotted for the three categories: Cellular component, Molecular function, and Biological process. Plotted data points are given in S6 Data. ABR, auditory brainstem response; ER, endoplasmic reticulum; GO, Gene Ontology; MGI, Mouse Genome Informatics; MGP, Mouse Genetics Project.

We carried out a GOSlim (high-level version of Gene Ontology) analysis to ask if the genes newly associated with hearing impairment (*n* = 38) showed a similar distribution of gene ontology features to the group of genes previously known to be involved in deafness (*n* = 362), and to compare them with all genes tested in this screen (*n* = 1,211) and a group of genes associated with ABR waveform defects (*n* = 27, described later). The novel genes showed nothing notably different to the full set of genes screened (Fig 2). However, nearly 70% of previously known genes had a Gene Ontology (GO) annotation for developmental processes, which was much higher than in the other groups analysed, suggesting an ascertainment bias for deafness due to early developmental defects in human and mouse. This finding suggests that our unbiased screen for new genes involved in hearing impairment at all levels of severity has revealed a fundamentally different class of genes compared with previously known genes underlying deafness.

Some of the 38 genes had links to existing pathways involved in deafness. For example, Duoxa2 is required for maturation and transport from the endoplasmic reticulum (ER) to the plasma membrane of Duox2, also known to underlie deafness through its role in hypothyroidism, leading to retarded cochlear development and impaired hearing [18]. Spns2 is a sphingosine-1-phosphate (S1P) transporter, and our discovery of its involvement in deafness supports the role of the S1P signaling pathway in hearing loss, alongside reports of *S1PR2* and *Sgms1* mutations causing deafness [19,20,21,22,23]. In contrast, many of the other genes discovered in this screen, such as *A730017C20Rik (Minar2)*, have no demonstrated role in a biological process and no a priori reason to predict they might be involved in deafness.

Although some of the 38 new genes identified by this screen showed strong expression in cochlear hair cells (S4 Table, column Q; https://gear.igs.umaryland.edu), in general expression levels did not show a strong correlation with our ABR findings.

### Progressive hearing loss

**Fig 3:**
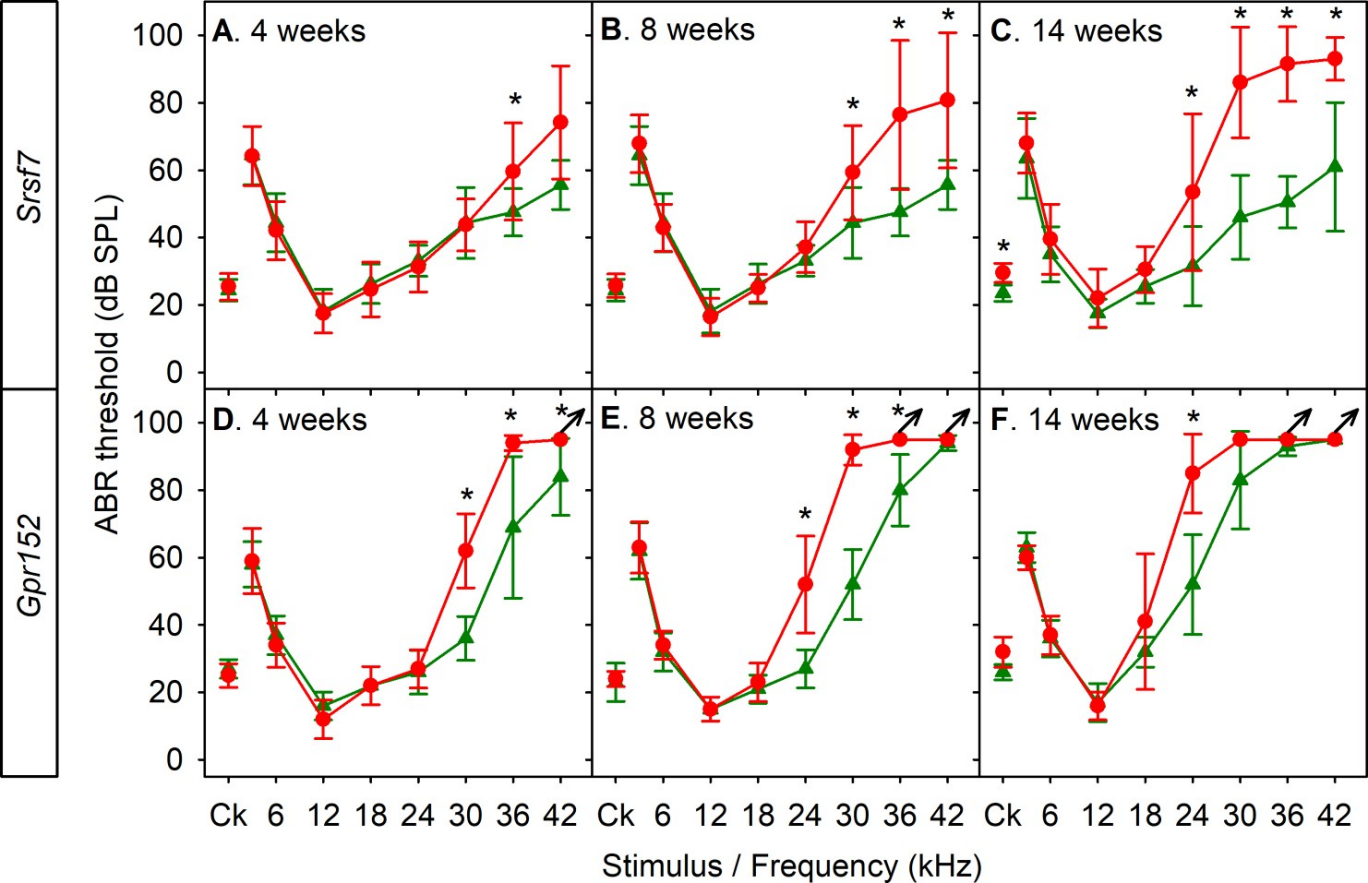
Progressive hearing loss in mutants. Several new mutant lines showed normal or near-normal ABR thresholds at young ages followed by progressive increases in thresholds with age. A-C. *Srsf7* heterozygous mutants. A. Four weeks old, wild-type littermates *n* = 9, *Srsf7* heterozygotes *n* = 12. B. Eight weeks old, wild-type littermates *n* = 3, *Srsf7* heterozygotes *n* = 7. C. Fourteen weeks old, wild-type littermates *n* = 10, *Srsf7* heterozygotes *n* = 10. D-F. *Gpr152* homozygous mutants. D. Four weeks old, wild-type littermates *n* = 5, *Gpr152* homozygotes *n* = 5. E. Eight weeks old, wild-type littermates *n* = 5, *Gpr152* homozygotes *n* = 5. F. Fourteen weeks old, wild-type littermates *n* = 5, *Gpr152* homozygotes *n* = 5. In all panels, means with standard deviations are plotted in red for mutants and green for wild-type littermate controls. Arrows indicate that there was no response, so the maximum sound level used was plotted. Asterisks indicate significant differences between mutants and wild-type controls (Mann–Whitney *U* test, *p* < 0.05). Plotted data points are given in S7 Data. ABR, auditory brainstem response; Ck, click; SPL, sound pressure level.

**Fig 4:**
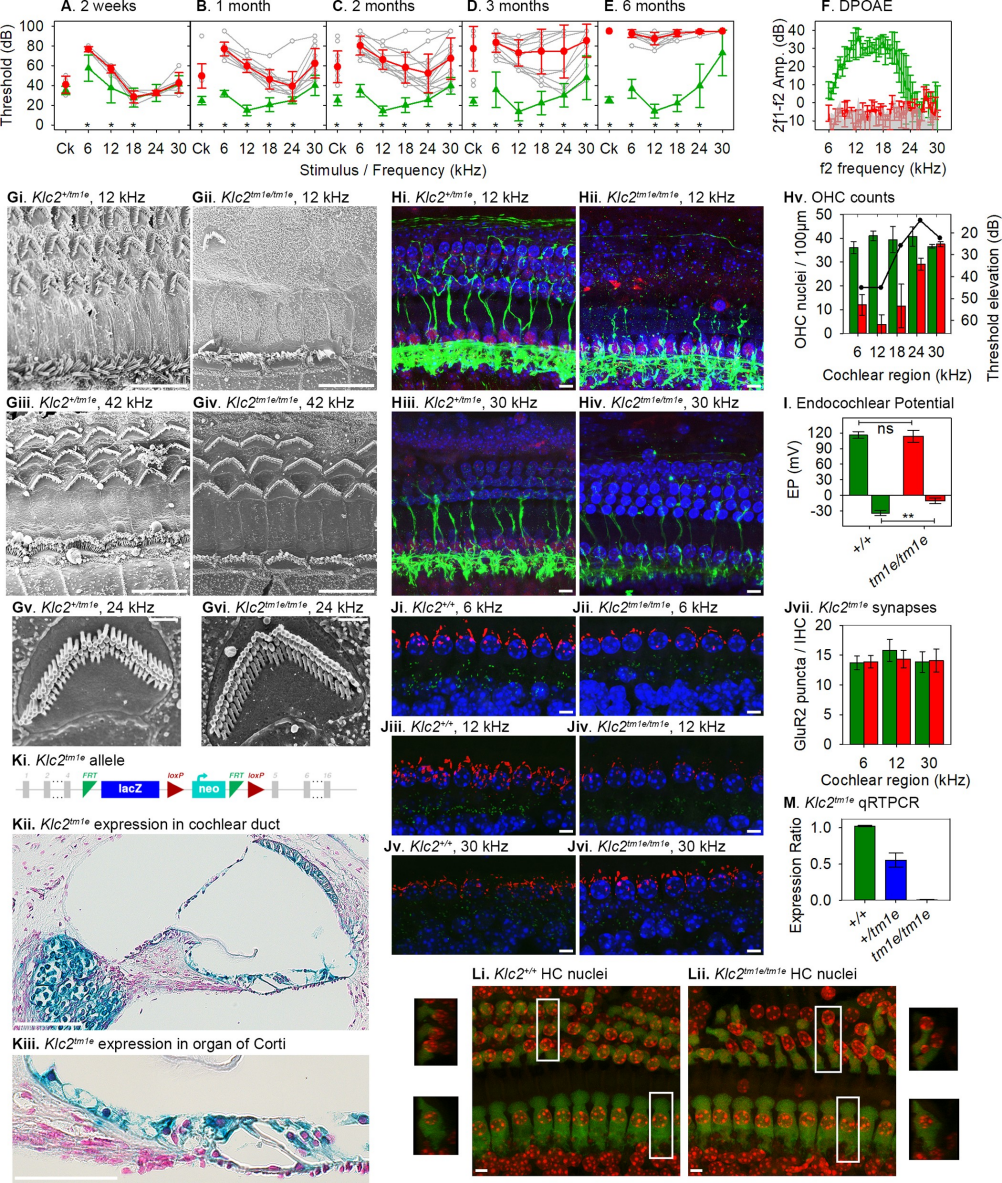
Pathology of the *Klc2* mutant. ABR thresholds of *Klc2* mutants are close to those of controls at 2 weeks old, and show significant, progressive increases in thresholds from one month onwards, mainly at low frequencies (3–18 kHz), increasing to affect higher frequencies by 6 months old (A-E) (wild types 2 weeks *n* = 4, 1 month *n* = 13, 2 months *n* = 13, 3 months *n* = 9, 6 months *n* = 15; *Klc2* homozygotes 2 weeks *n* = 6, 1 month *n* = 13, 2 months *n* = 13, 3 months *n* = 13, 6 months *n* = 18; Mann–Whitney *U* test, at 2 weeks old, *p* = 0.12 overall but *p* = 0.019 at 3 kHz and *p* = 0.01 at 6 kHz when testing stimuli separately; *p* < 0.001 at each later age shown by asterisks). F. DPOAEs at 6 months old show mutant amplitudes (red, *n* = 3) at the noise floor (grey) across all frequencies tested; wild types (green, *n* = 3) show normal emission amplitudes. Gi-vi. Scanning electron microscopy revealed extensive loss of OHC hair bundles at P28 in the cochlear regions, corresponding to the worst thresholds in mutants (12 kHz; Gi. heterozygote *n* = 4, Gii. homozygote *n* = 11), while there was little sign of hair cell loss at higher-frequency regions (42 kHz; Giii. heterozygote, Giv. homozygote). Remaining hair bundles had a normal appearance (Gv. heterozygote, Gvi. mutant). Scale bars, g-j, 10 μm; k-l, 1 μm. Hi-iv. Confocal imaging at P28 showed that many OHC nuclei were missing in the most affected regions (12 kHz; Hi. heterozygote *n* = 4, Hii. homozygote *n* = 5), but most hair cell nuclei were present at less affected regions (30 kHz; Hiii. heterozygote, Hiv. homozygote). Blue, DAPI-labelled nuclei; red, CtBP2-labelled ribbons and IHC nuclei; green, neurofilament-labelled unmyelinated dendrites. Scale bars, 10 μm. Hv. Quantification of OHC nuclei from confocal images demonstrated significant reduction in mutants (red) at best-frequency regions from 6 to 24 kHz and no significant difference with controls (green) at the 30-kHz region. Black line represents ABR threshold elevation in mutants compared with littermate controls. I. EPs in wild types (green) and homozygotes (red) show no significant difference in mutants (homozygotes 113.8 ± 11.5 mV, *n* = 10; wild-type littermates 116.0 ± 6.5 mV, *n* = 10; *t* = 0.518, df = 14, two-tailed *p*-value = 0.613). Maximum negative potentials during anoxia are significantly reduced in homozygotes (lower part of plot) (homozygotes −10.0 ± 5.39 mV, *n* = 6; wild-type littermates −33.7 ± 4.8 mV, *n* = 6; *t* = −8.050, df = 10, two-tailed *p*-value = 0.0000111). Ji-vi. Confocal imaging of IHCs at P28 showed no obvious abnormalities of GluR2-labelled postsynaptic densities (green), but less extensive Kcnma1-labelled patches (red) of IHCs in mutants compared with controls at the frequency regions showing the worst thresholds (6 and 12 kHz) (*n* = 7 homozygotes, 5 littermate controls). Scale bars, 5 μm. Jvii. Counts of green-labelled GluR2 puncta per IHC show no difference between mutants and wild types (6 kHz, homozygotes *n* = 7, wild types *n* = 5, *t* test, *p* = 0.7422; 12 kHz, homozygotes *n* = 7, wild types *n* = 5, *t* test, *p* = 0.0737; 30 kHz, homozygotes *n* = 5, wild types *n* = 6, *t* test, *p* = 0.7089). Ki. Representation of the allele, with exons in grey, FRT sites in green, loxP sites in red, and lacZ and neo components of the inserted construct labelled. Kii-iii. Expression of *Klc2* in the cochlear duct of a heterozygote (*n* = 3) using the *LacZ* reporter system in the allele. Blue-labelled areas show expression in cells surrounding the cochlear duct and spiral ganglion. Kiii shows a higher magnification of the organ of Corti. Scale bar on Kii, 100 μm, on Kiii, 50 μm. Li-ii. Confocal images of the organ of Corti in a wild type (left) and homozygote (right) at P28 labelled with Myo7a antibody (false-coloured green) showing hair cell bodies and DAPI (false-coloured red) showing nuclei. Small images at left and right show the areas marked in white boxes rotated through 90° around the radial cochlear axis to show a mid-modiolar view of the hair cells. Nuclei appear in similar locations in mutants and controls (base of OHCs, middle of IHCs). Scale bars, 5 μm. M. qRT-PCR of *Klc2* mRNA from brain at P28 showed complete knockdown of transcript in homozygotes (red, *n* = 3); heterozygotes (blue, *n* = 4) showed around half of the wild-type level (green, *n* = 2). All plots are means ± standard deviation. Plotted data points are given in S8 Data. ABR, auditory brainstem response; DPOAE, distortion product otoacoustic emission; EP, endocochlear potential; FRT, flippase recombinase target; IHC, inner hair cell; *lacZ*, gene encoding β-galactosidase; loxP, locus of crossover in P1 bacteriophage; OHC, outer hair cell; P28, postnatal day 28; qRT-PCR, quantitative real-time PCR.

As our goal in carrying out the screen was to identify new genes involved in adult-onset hearing loss, we carried out recurrent ABR recordings (usually at 4, 8, and 14 weeks old, plus shortly after the normal onset of hearing at 2 and 3 weeks old if thresholds were raised at 4 weeks) on some of the mutant lines. Remarkably, several of the mutants we studied showed relatively normal early development of ABRs followed by progressive increase in thresholds. These included *Srsf7* heterozygotes (encoding a splicing factor; Fig 3A–3C), *Gpr152* homozygotes (a G-protein–coupled receptor; Fig 3D–3F), and *Klc2* (Fig 4A–4E), plus *Spns2* [11], *Wbp2* [10], *Acsl4*, *Zfp719*, *Ocm*, and *Klhl18* homozygotes.

The finding of multiple new genes underlying progressive hearing loss and/or impairment of responses to high frequencies (Figs 1 and 3) is important because progressive hearing loss in humans is very common and often affects high frequencies first, yet we have few clues to the pathological molecular processes involved. The mouse alleles studied here are relatively severe in their effect on protein expression, but variants in the human population may have milder effects on protein function and lead to later onset of hearing loss. Importantly, the finding of genes involved in normal development but later deterioration of hearing identifies molecular pathways likely to underlie adult-onset progressive hearing loss in humans.

### Varied pathophysiology underlying deafness

We analysed further a subset of mutant lines and revealed a wide range of pathological conditions underlying hearing impairment. Two examples of contrasting phenotypes are the *Klc2* and the *Ywhae* mutant lines.

*Klc2* mutants showed a progressive increase in ABR thresholds with age, mostly affecting low frequencies (Fig 4A–4F), with a sensorineural (not conductive) pathology. *Klc2* encodes kinesin light chain 2, which, together with kinesin heavy chains (encoded by *Kif5*), forms the kinesin-1 motor complex, a microtubule-associated anterograde transporter. The allele design (Fig 4Ki) allowed us to use the gene encoding β-galactosidase (*LacZ*) as a reporter system, showing that *Klc2* was expressed in the epithelial cells lining the cochlear duct and strongly in the spiral ganglion (Fig 4Kii-iii). The middle ear and gross structure of the inner ear appeared normal. The endocochlear potential (EP) was maintained at a normal level even up to 6 months of age, but the anoxia potential in scala media was significantly less negative in these mutants, consistent with loss of hair cell conductance (Fig 4I). At one month old, there was extensive loss of outer hair cell (OHC) hair bundles (Fig 4Gi-vi), DAPI-stained OHC nuclei, and CtBP2-labelled presynaptic ribbons of OHCs primarily in the region that normally responds best to 12 kHz (60%–70% of the distance along the cochlear duct from the base), corresponding to the worst ABR thresholds (Fig 4Hi-v). There were few signs of inner hair cell (IHC) degeneration, but the increase in threshold was larger than would be expected if only OHCs were affected, suggesting IHC dysfunction. Klc2 is involved in anterograde transport of PI3K, which mediates insertion of AMPA receptors at synaptic membranes [24] and GluR1/2-containing vesicles to axon terminals [25,26]. We found no abnormality in GluR2-labelled postsynaptic densities below mutant IHCs (Fig 4Ji-vii), suggesting other transport systems must move this AMPA receptor to the membrane. Klc2 also interacts with Kcnma1, the calcium-activated potassium channel (BK channel) that underlies the *I*_K,f_ current required for very rapid responses of IHCs and contributes to protective efferent suppression of OHCs [27,28,29,30]. We found that labelling of Kcnma1 in IHCs was less extensive in mutants compared with wild-type controls (at 12kHz; Fig 4Ji-vi), implicating Kcnma1 in the pathological mechanism; however, knockout of *Kcnma1* leads to less severe loss of thresholds [31], so this alone cannot explain the extent of dysfunction in the *Klc2* mutants. Finally, kinesin-1 has been implicated in maintenance of the hair cell nucleus in its correct position by interacting with Nesp4 in the outer nuclear membrane [32,33], and *Nesp4* mutations lead to location of the OHC nucleus at the top of the cell and subsequent degeneration [34]. We did not find mislocalisation of OHC or IHC nuclei (Fig 4Li-ii), suggesting this was not the mechanism underlying hearing loss in the *Klc2* mutants, and that redundancy between kinesin light chains may compensate for loss of Klc2 in nuclear localisation. No disease-associated loss-of-function mutations of human *KLC2* have been reported yet to compare with the *Klc2* mutant mice, which show a complete lack of *Klc2* mRNA (Fig 4M). However, a human gain-of-function *KLC2* mutation (216-bp deletion upstream of the coding region) leading to increased *KLC2* expression causes spastic paraplegia, optic atrophy and neuropathy (SPOAN), a neurodegenerative disorder involving progressive axonal neuropathy [35].

**Fig 5:**
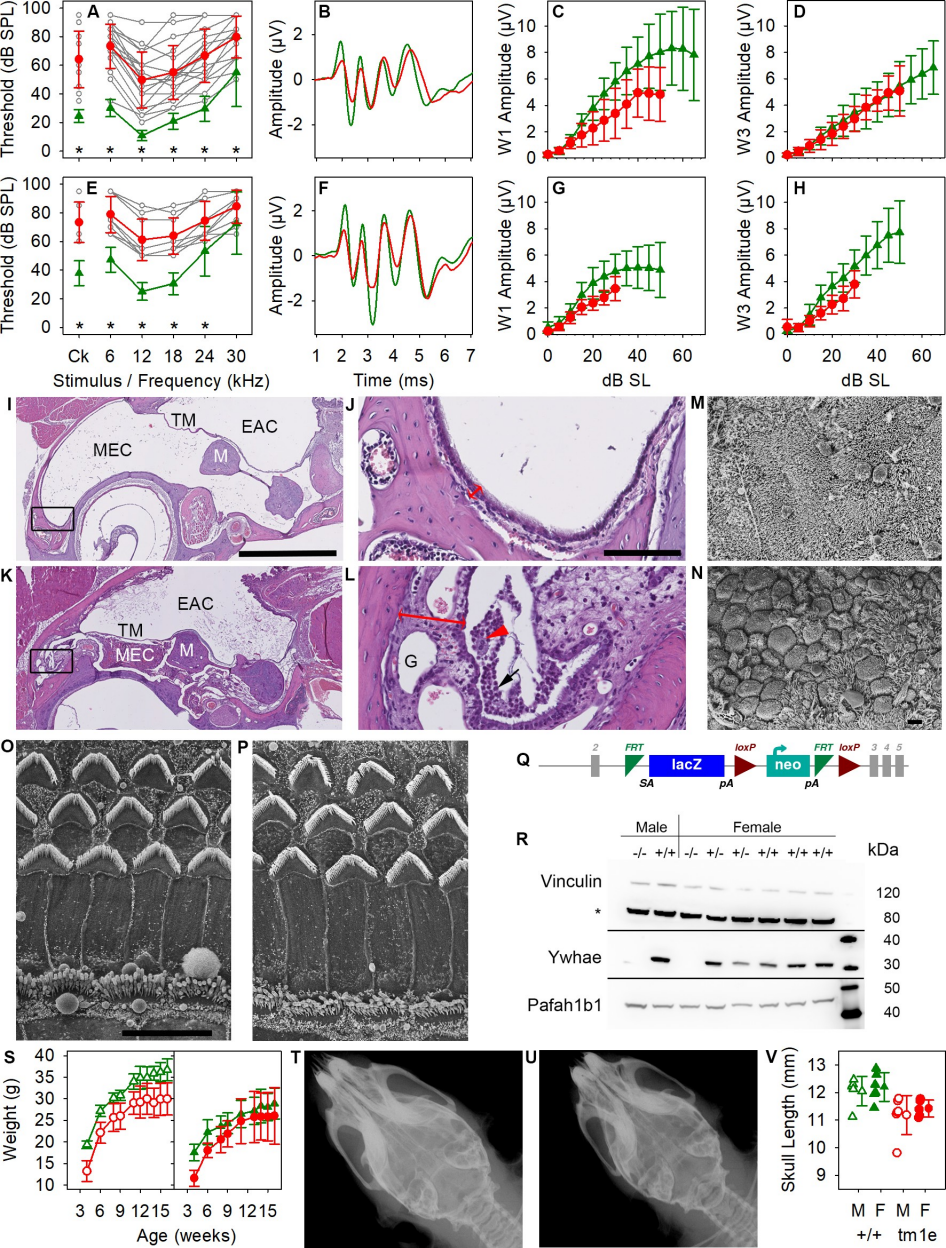
Pathology of the *Ywhae* mutant. ABR thresholds were significantly raised at 8 weeks old in mutants (A,E) (Mann–Whitney *U* test, *p* < 0.001) but were highly variable between individuals (grey lines), while click-evoked ABR waveforms were close to normal (B,F show group mean waveforms at 20 dB above threshold), and reduced but nonsignificant amplitude growth with increasing stimulus level in mutants (C,D,G,H) (Mann-Whitney-Wilcoxon test with Holm correction for multiple testing). *Ywhae* homozygotes (red) and wild-type littermates (green) with individual mutant thresholds (grey) on a 50% C57BL/6N, 50% 129S5 (A-D) (*n* = 18 homozygotes, 12 wild types for thresholds; *n* = 10 homozygotes, 11 wild types for amplitude functions) or a 12.5% C57BL/6N, 87.5% 129S5 genetic background (E-H) (*n* = 9 homozygotes, 13 wild types for thresholds; *n* = 6 homozygotes, 13 wild types for amplitude functions). Heterozygotes have normal ABR thresholds. (I-L) Coronal sections of the middle ear in wild type (I,J) and homozygous *Ywhae* mutants (K,L). The tympanic membrane is retracted in the mutant, and the middle ear contains inflammatory debris. The mucosa is thicker in the mutant (double-headed red arrow), containing granulation tissue (‘G’), infiltrated immune cells (black arrow in L), and foamy macrophages (red arrowhead). Black rectangles in I and K indicate the areas enlarged in J and L. Scale bar for I and K is 1 mm; in J and L it is 100 μm. *n* = 2 homozygotes, 2 heterozygotes, 4 wild types. M,N. Scanning electron micrographs of the middle ear epithelium near the opening of the Eustachian tube in wild-type (M) and homozygous *Ywhae* mutant (N), showing widespread goblet cells and fewer ciliated epithelial cells in the mutant compared with the wild type, which is rich in ciliated cells. Scale bar is 10 μm. *n* = 5 homozygotes, 5 heterozygotes, 3 wild types. O,P. Scanning electron micrographs of the organ of Corti in a heterozygote (O) and homozygous mutant (P) showing normal appearance. OHCs are shown at the top, each with a V-shaped stereocilia bundle, and IHCs at the bottom. Images taken from 60% of the distance along the cochlear duct from the base. *n* = 7 homozygotes, 5 heterozygotes, 2 wild types. Scale bar is 10 μm. Q. Design of the *Ywhae*^*tm1e(EUCOMM)Wtsi*^ mutation with exons in grey, FRT sites in green, loxP sites in red, and *lacZ* and neo components of the inserted construct labelled. R. Western blot of Ywhae and Pafah1b1 (Lis1) protein in brain showing no detected Ywhae in homozygous mutants (−/−), while expression of Pafah1b1 (encoded by a nearby gene) was unaffected. Vinculin was used as a loading control. *n* = 2 homozygotes, 2 heterozygotes, 4 wild types. S. Body weight growth with age in males (left) and females (right), showing significantly reduced weights in homozygotes (red) compared with wild types (green) (males, *n* = 7 homozygotes, 6 wild types; females, *n* = 7 homozygotes, 7 wild types; mixed model framework test as described by Karp and colleagues [42] at 4 weeks, 16 weeks, and area under the curve, *p* = 6.9 × 10^−3^, 0.013, and 6.9 × 10^−3^ respectively). T-V. X-rays of wild type (T) and homozygotes (U) showing shorter skull length (V) in the mutants (*p* = 3.9 × 10^−5^; *n* = 6 male and 5 female homozygotes and 5 male and 6 female wild types; mixed model framework test, males and females *p* = 3.9 × 10^−5^). All plots are means ± standard deviation. Plotted data points are given in S9 Data. ABR, auditory brainstem response; Ck, click; EAC, external auditory canal; FRT, flippase recombinase target; IHC, inner hair cell; *lacZ*, gene encoding β-galactosidase; loxP, locus of crossover in P1 bacteriophage; M, malleus; MEC, middle ear cavity; OHC, outer hair cell; SL, sensation level; SPL, sound pressure level; TM, tympanic membrane.

In contrast, the *Ywhae* mutants showed increased thresholds across all frequencies associated with variable amounts of accumulated fluid and exudate containing inflammatory cells in the middle ear, suggesting predisposition to otitis media (Fig 5A–5M). The middle ear mucosa appeared thickened with granulation tissue in sections (Fig 5L), and scanning electron microscopy of the luminal surface showed an open Eustachian tube in mutants, but abundant clusters of goblet cells (presumed to produce mucus) with fewer ciliated epithelial cells, which would normally contribute to the clearing of excess mucus (Fig 5M and 5N). The variability in thresholds between individual *Ywhae* mutants, relatively flat increase across all frequencies, and near-normal ABR waveform support a conductive hearing loss (Fig 5A–5H). The surface of the organ of Corti looked normal (Fig 5O and 5P) but we cannot exclude a sensorineural component in some of the more severely affected mutants, possibly due to the impact of 129S5-derived alleles in the mixed genetic background, or an effect of persistent inflammation of the middle ear [36]. Ywhae, also known as 14-3-3ε, is a member of the highly conserved 14-3-3 phosphoserine/threonine binding family, which have many interacting partners and are thought to provide a scaffold allowing coordination of intracellular signaling [37,38]. Ywhae is normally widely and strongly expressed throughout the body [39] and within the cochlea [40], and the mutation led to an absence of detectable Ywhae protein in homozygotes (Fig 5R). Not surprisingly, *Ywhae* mutants showed a number of other abnormal phenotypes similar to those reported in another *Ywhae* mutant [41] (see S1 Table), including reduced viability of heterozygotes on a C57BL/6N background (20 heterozygotes out of 192 offspring from heterozygote × wild-type matings at weaning, chi- squared *p* < 0.0001) and homozygotes on a mixed C57BL/6N and 129S5/SvEvBrd/Wtsi background (53 homozygotes at 2 weeks old from 685 offspring from heterozygous intercrosses, chi-squared *p* < 0.0001). *Ywhae* homozygotes also showed reduced growth (Fig 5S) and a shortened skull (Fig 5T–5V). Craniofacial malformations may affect Eustachian tube structure and function, leading to otitis media, but there are many other possible pathological mechanisms not yet explored. Similar fluid-filled middle ear and conductive hearing loss phenotypes were found in the *Mcph1* mutant [12] and *Slc25a21* mutants with reduced *Pax9* expression [8].

A third distinct pathology found was a reduction in EP. Normally, a high resting potential in the cochlear endolymph is generated by the stria vascularis. This is necessary for normal sensory hair cell function. Progressive disorganisation of the stria vascularis accompanies the reduced EP in *Spns2* mutants [11]. A fourth example is the *Wbp2* mutant, in which abnormal structure of synapses between IHCs and cochlear neurons and swelling of nerve terminals leads to progressive increase in ABR thresholds [10].

The finding of a wide range of primary pathological processes in these mouse mutants as outlined above suggests that the pathogenesis of hearing loss in the human population may be equally heterogeneous. The limited information gleaned from human temporal bone studies supports the suggestion of heterogeneous pathophysiology underlying progressive hearing loss [43].

### Other features associated with deafness in the new mutants

Many mouse mutants with deafness were originally detected, because they also had a balance defect leading to circling and/or head bobbing; thus, many of the earliest genes to be identified were those involved in early developmental problems such as gross inner ear malformations or sensory hair cell developmental abnormalities affecting both the cochlea and vestibular part of the inner ear. It is notable that none of the 38 new mutant genes we report here showed any sign of leading to a balance defect (S1 Table). Nine lines had reduced viability assessed at postnatal day 14, with three of these lines producing so few homozygotes that heterozygotes were passed through the phenotyping pipelines instead (*Brd2*, *Srsf7*, and *Setd5*). Six lines had either male or female infertility (*Pex3*, *Mkrn2*, *Herc1*, *Camsap3*, *Mcph1*, *Usp42*), which is higher than the expected 5% based on larger panels of mutant alleles. Corneal or lens defects were observed in five lines (*Spns2*, *Pex3*, *Agap1*, *Mcph1*, *Usp42*). Occurrence of anomalous features in other systems tested were generally scattered across mutant lines and phenotypes, with *Duoxa2* and *Ywhae* mutants showing the largest number of other abnormalities (S1 Table).

### Mutant lines with normal thresholds but abnormal waveforms

**Fig 6:**
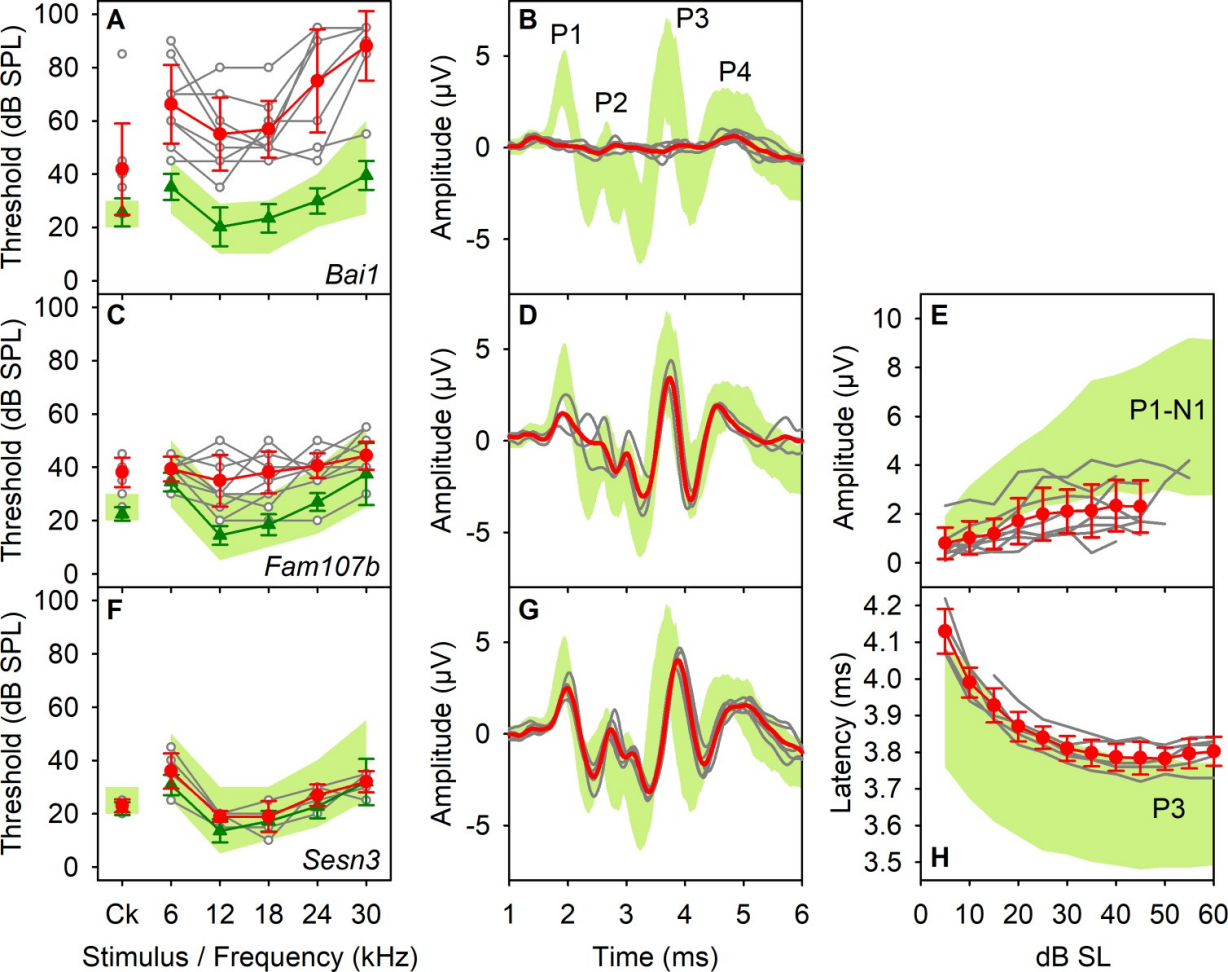
Genes affecting central auditory system function. Examples of three mutant mouse lines with altered ABR waveforms are shown. ABR thresholds are plotted in the left column, click-evoked ABR waveforms recorded at 50-dB SL in the second column, and IOFs (parameter versus stimulus level above threshold, dB SL) are plotted in the third column. The green band denotes the 95% reference range of control values (defined in Methods). Red lines and circles represent mean responses (±SD) from mutant mice. Responses from individual mutants are shown by grey lines and open circles. Green lines and triangles represent the mean thresholds (±SD) for control mice recorded in the same week as the mutants. A,B. The *Bai1 (Adgrb1)* mutant line produced ABRs that were grossly abnormal. C-E. The *Fam107b* mutants produced a mild increase in thresholds but also have ABR waveforms with significantly reduced wave 1 amplitude. F-H. *Sesn3* mutants showed normal thresholds but prolonged P3 latency. Plotted data points are given in S10 Data. ABR, auditory brainstem response; IOF, input-output function; SL, sensation level; SPL, sound pressure level.

By analysis of click-evoked ABR waveforms, we identified 27 additional mutant lines with normal hearing sensitivity, but which had abnormal patterns of neural responses, such as smaller ABR wave amplitudes or prolonged latencies, determined using objective criteria (S1 Table; Fig 6; S3 Fig; S3 Data). Ten further mutant lines from the 38 with ABR threshold elevation also exhibited abnormal ABR waveform shapes (S1 Table; S4 Fig; S4 Data). The ABR waveform is a complex mixture of voltage changes reflecting the sum of excitatory and inhibitory activity at different times after stimulus onset and different physical locations within the brain relative to the position of the recording electrodes. Wave 1 reflects auditory nerve activity, and later waves reflect activity higher up the central auditory pathways. Some mutant lines had reduced wave 1 amplitudes (Fig 6; S3 and S4 Fig; S4 Data; S1 Table), which may result from desynchronisation of the onset of firing in auditory nerve fibres [44], or reduced numbers of auditory nerve fibres contributing to the ABR, or a selective loss of high spontaneous rate/low threshold neurons, which have maximal discharge rate at stimulus onset, or inefficient synaptic recovery during the short gap (23.5 ms) between stimuli. Other mutants showed abnormal amplitudes or latencies of later waves, suggesting auditory processing anomalies in the central auditory system (Fig 6; S3 and S4 Figs; S3 and S4 Data). These changes could reflect abnormal inherent excitability of auditory neurons or an alteration in the balance of excitatory and inhibitory inputs onto these neurons, resulting in increased or decreased discharge or synchrony. Mutants showing changes in latency are most easily explained by changes in neural conduction speed, alterations in synaptic delays, or changes in the relative contributions of different components in this complex neuronal pathway. Finally, in the most extreme example (*Bai1*, also known as *Adgrb1*), the mice exhibited clear auditory-evoked responses and measurable thresholds, but the ABRs were so abnormal that it was not possible to determine the equivalent peaks to quantify and compare with control mice (Fig 6). This set of 27 mutants with waveform anomalies will be an interesting group to analyse further, because central auditory function is critical for normal sound perception. Such deficits may translate in humans to altered performance in sound localisation, ability to follow salient acoustic stimuli in background noise, discrimination of specific speech features, and other auditory processing disorders (e.g., [45]).

### Conclusions

We used a rapid ABR protocol to carry out a high-throughput screen of 1,211 new mouse mutants and revealed a new spectrum of functional deficits in hearing that would not have been detected using simpler screens, such as the startle response. These include mild-moderate degrees of hearing impairment, frequency-specific impairments (low or high frequencies), and a group with abnormal ABR waveforms that likely have deficits in central auditory pathways. In a subset of the new mutant lines, we have examined other ages to establish the time course of hearing loss and investigate the pathophysiological mechanisms underlying the raised ABR thresholds. A broad range of pathologies was found, and many mutants showed normal development followed by progressive hearing loss. We have shown that some of the genes highlighted by this study play a role in human hearing, including 2 genes with mutations that can account for recessive deafness in families and 11 genes that are associated with variation in auditory thresholds in the UK 1958 British Birth Cohort cross-sectional population. Thus, mouse mutants can be an effective means to identify candidate genes for human deafness.

This project has provided insights into the wide range of pathological processes involved in hearing impairment and has revealed a surprising number of unexpected genes involved in deafness, suggesting extreme genetic heterogeneity. For this reason, it is likely that therapies will need to be directed at common molecular pathways involved in deafness rather than individual genes or mutations. Each new gene identified gives insight into the metabolic pathways and regulatory processes involved in hearing and thus provides a rich source of targets for development of therapies for the restoration of hearing.

## Materials and methods

### Ethics statement

Mouse studies were carried out in accordance with UK Home Office regulations and the UK Animals (Scientific Procedures) Act of 1986 (ASPA) under UK Home Office licences, and the study was approved by the King’s College London and Wellcome Trust Sanger Institute Ethical Review Committees. Mice were culled using methods approved under these licences to minimise any possibility of suffering.

For human studies, informed consent was obtained from the adult participants and the parents or guardians of children prior to participation, and the experiments conformed to the principles set out in the WMA Declaration of Helsinki and the Department of Health and Human Services Belmont Report. The US patient provided consent for clinical whole exome analysis and written consent for inclusion as a case report. Testing was conducted during the routine clinical care of a patient in the US; thus, in accordance with US law, this study is exempt from Institutional Research Board approval.

### Generation of mice

Mutant mouse lines were generated using targeted mutations in mouse ES cells [5,6]. The viability of new mutants was determined by genotype distribution at weaning. When possible, mice homozygous for the targeted mutation were used for screening. If a mutation proved to be embryonic lethal or had significantly reduced viability at weaning, heterozygous mice were used instead (356 genes out of 1,211; 29.4%). In most cases, the knockout first conditional-ready (*tm1a*) allele for each gene was used, but a subset of the genes were tested using the derived *tm1b* allele, which had deletion of a critical exon(s), or other mutations (S2 Table, column B). The *tm1a* allele is designed to knock down transcription by introducing a large cassette into the gene, but not all genes were completely inactivated (see S2 Table and column X in [5], for some examples). Most mutant mice were screened on a C57BL/6 genetic background. This included some lines on a mixed C57BL/6Brd-*Tyr*^*c-Brd*^; C57BL/6N line and others on a pure C57BL/6N line (S1 and S2 Tables, column D). Mutant data were compared with a large set of wild-type data on the same genetic background. When mice were screened on mixed genetic backgrounds (for example, *Ywhae*^*tm1e*^), age and strain-matched wild-type mice were used alongside the mutants. Positive control lines known to have a hearing impairment were compared with their littermate controls on the same, varied genetic backgrounds (S1 Table). At the age screened, 14 weeks, we found no apparent effect of the known *Cdh23*^*ahl*^ allele carried by C57BL/6 mice on ABR thresholds [46], and mutant responses were compared with mice of the identical genetic background. The *Cdh23*^*ahl*^ allele may have interacted with any of the new mutations to exacerbate their effect, such that the phenotype was easier to detect at 14 weeks, so this screen could be regarded as a sensitised screen [47].

### Phenotyping pipelines

Details of the full phenotyping pipelines in the Mouse Genetics Project (MGP) have been reported elsewhere [5]. Two assays required mice to be immobilised and so to minimise stress to the mice, ABR testing was performed under anaesthesia immediately before the DEXA/Faxitron assays. Balance was assessed by observation of gait, head bobbing or circling, the rotarod test, or contact righting test. ABR testing was performed on three phenotyping pipelines used over successive time periods, termed MGP Pipeline 2, Mouse GP, and MGP Select, respectively. ABRs were recorded in mice aged 13 weeks (±3 days) on MGP Pipeline 2, and at 14 weeks (±3 days) on Mouse GP and MGP Select pipelines. Mice were maintained on a normal lab chow diet on MGP Pipeline 2 and MGP Select pipelines, but on a high-fat lab chow for the Mouse GP pipeline.

### ABR recordings

ABRs were recorded using the methods described in detail in [4]. Mice were anaesthetised using intraperitoneal ketamine (100 mg/kg, Ketaset, Fort Dodge Animal Health, KS) and xylazine (10 mg/kg, Rompun, Bayer Animal Health), or a 10% greater dose for the MGP Select pipeline. Mice were placed on a heating blanket inside a sound-attenuating booth. Subcutaneous needle electrodes were inserted in the skin on the vertex (active) and overlying the ventral region of the left (reference) and right (ground) bullae. Stimuli were presented as free-field sounds from a loudspeaker whose leading edge was 20 cm in front of the mouse’s interaural axis. The sound delivery system was calibrated using an ACO Pacific 7017 microphone. For threshold determination, custom software and Tucker Davis Technologies hardware were used to deliver click (0.01-ms duration) and tone pip (6, 18, 24, and 30 kHz of 5-ms duration, 1-ms rise/fall time) stimuli over a range of intensity levels from 0 to 95 dB sound pressure level (SPL, re. 5 μPa) in 5-dB steps. Averaged responses to 256 stimuli, presented at 42.6/s, were analysed and thresholds established as the lowest sound intensity giving a visually detectable ABR response (S1 Fig; S1 Data). Following completion of recording, mice were injected with intraperitoneal atipamezole (1 mg/kg, Antisedan, Pfizer) to promote recovery.

A fixed recording protocol was followed:

1. A series of click-evoked ABRs were recorded, ranging from 0 to 85 dB SPL in 5-dB intervals.

2. Tone-evoked ABRs were recorded for a fixed set of frequencies (6, 12, 18, 24, and 32 kHz) over sound levels ranging from 0 to 85 dB SPL in 5-db intervals. Different SPL ranges were recorded for different test frequencies to improve the time efficiency of the recording process (6 kHz, 20 to 85 dB; 12 kHz, 0 to 70 dB; 18 kHz, 0 to 70 dB; 24 kHz, 10 to 70 dB; 30 kHz, 20 to 85 dB). Responses were recorded in an array, beginning with the lowest stimulus level, in decreasing frequency order before stepping up to the next (5 dB higher) stimulus level. If mice appeared to have hearing impairment, the upper limit of SPLs was extended to 95 dB for each test frequency and for clicks (representing the upper limit of the linear range of our sound system at these frequencies).

For the ABR screen, we aimed to test a minimum of four mutant mice per line (of either sex). For other tests on the pipeline, 14 mutant mice (7 males and 7 females) were required. Phenotyping cohorts were issued as mice became available, such that several partial cohorts were issued, to achieve the required number of 14 mice for each single line. This allowed the ABR assay to pick up further mice from any lines that exhibited any features of interest to extend the number tested beyond the target of four. In addition to mutant mice, at least four wild-type mice from the same matings used to generate the mutants were tested each week from each core genetic background of the mutants tested. These wild-type results formed a local control group for comparison with the mutant lines and also contributed to a large reference range of control data that were used to determine if ABR results from a particular mutant line were significantly abnormal.

We compared ABR thresholds measured in 1,142 wild-type mice (female, *n* = 583; male, *n* = 559) from the pure C57BL/6N or mixed C57BL/6J and 6N genetic backgrounds. No sex differences were noted for mice of either genetic background (S2 Fig U-X; S2 Data).

### Data analysis

We compared mutant data to a fixed population of control mice of the same genetic background (Pipeline 2, C57BL/6J and 6N mixed *n* = 201; Mouse GP, C57BL/6J and 6N mixed *n* = 951; MGP Select, pure C57BL/6N *n* = 742). The reference range for each parameter was defined as a 95% range of control values, from the 2.5 percentile to the 97.5 percentile of the control population, shown as a distribution around the median of the control data.

ABR responses were considered in three phases of analysis as follows:

**1. ABR threshold and hearing sensitivity.** For each stimulus used (click and five tone frequencies), ABRs recorded over the range of sound levels tested were plotted as a stack, ordered by increasing dB SPL (S1 Fig; S1 Data). Threshold (dB SPL) was estimated by visual inspection of the stacked ABR traces as the lowest sound level at which any component of the ABR waveform was recognisable and consistent with responses recorded at higher sound levels, taking into account the characteristic lengthening of peak latency as threshold is approached. Thresholds for each stimulus were plotted to give a profile of the hearing sensitivity of each mouse.

**2. Waveform shape comparisons.** Through consistent, reproducible electrode placements, it was possible to compare, qualitatively and quantitatively, the waveform shapes of click-evoked ABRs. Wave 1 is understood to reflect auditory nerve activity, but as the responses represent a complex mixture of responses detected at a single point, there is some uncertainty in ascribing specific brain locations to specific features of the ABR waveform. The free-field binaural stimulation conditions we used complicates interpretation further, because there are binaural interactions even within the cochlear nucleus, e.g., [48]. The ABR represents the summed electrical vectors detected by the electrodes as synchronised action potential volleys (particularly from onset-responding neurons) traverse the central auditory pathways. As these pathways can be both excitatory and inhibitory, as well as both ascending and descending, and are distributed in a 3D volume, interpretation is complex.

Waveforms recorded to clicks at 20 dB and 50 dB above threshold (sensation level [SL]) were plotted for mutant and control mice, along with an average of the ABR amplitude over time across mice for each genotype. In these responses, we could identify four waves (positive to negative deflections; S1 Fig; S1 Data). We first performed a qualitative comparison of click-evoked waveforms recorded at 50-dB SL (Fig 6; S4 Fig; S4 Data). The averaged mutant waveforms were compared with both the averaged control waveform and a 95% reference range of waveform amplitudes. We also compared individual mutant responses with the reference range. If both comparisons were in agreement between at least two of three experienced observers, a quantitative analysis was carried out of the peak amplitude, latencies, and intervals of these waveforms, by measurement of input-output functions (IOFs).

**3. IOFs.** Using click-evoked ABRs, waveforms were analysed in detail to determine the amplitude and latency of positive and negative peaks of the waveform at each stimulus level recorded (S1 Fig; S1 Data). This was performed using software routines developed by Brad Buran and kindly donated for our use by M.C. Liberman (Harvard University). We found wave 1 and wave 3 were highly consistent in control mice. However, whilst wave 2 was clearly present as a single peak at low sound levels, it often split into two components at higher sound levels, making analysis complicated, so we did not include it. From these measures, we calculated the peak-peak amplitude of waves 1, 3, and 4, the amplitude of the N2-P3 component, and the intervals from P1 to P3 and N1 to N3. IOF curves were plotted relative to click threshold for each mouse (i.e., parameter plotted against dB SL). IOFs of individual mice (mutants and local controls) were plotted, together with 95% reference range generated for each parameter for controls.

### Significance calls on mutant data

Wild-type control mice of the same genetic background tested in the same week as mutants were used as a local control population. As mutant mice were often tested in separate weeks to obtain the required numbers, the local control population for each mutant line varied in numbers. We compared each mutant population of results with a 95% reference range obtained from a fixed large number of wild-type controls of the same genetic background. Control populations for ABR thresholds of C57BL/6N or mixed C57BL/6N and 6J mice were not normally distributed (Shapiro-Wilk Normality test, *p* < 0.001). Furthermore, the large disparity in population sizes of the groups invalidates the use of traditional statistical tests giving *p*-values (e.g., *t* tests or analysis of variance) [42]. Thus, we used the following criteria to define parameters considered to be abnormal compared with controls.

**1. ABR thresholds.** A mutant line was considered to have abnormal ABR thresholds if one of two criteria were met: (1) at least 60% of mutant mice had thresholds for any stimulus outside the reference range, or (2) if the mean mutant threshold for any stimulus was at least 20 dB different from the median of the reference range for that stimulus. Thus, thresholds could be considered abnormal if they were elevated above controls (lower sensitivity) or reduced below controls (enhanced sensitivity). We did not find any mutants with enhanced sensitivity.

**2. Waveform shape.** Click-evoked ABR waveform shapes were compared at 20 dB and 50 dB SL. These comparisons were used as a subjective triage step in the assessment of whether waveform shapes were normal or perturbed in responses from mutant mice. A dataset was considered potentially interesting if two experienced observers considered the waveforms to be perturbed. In these cases, peak amplitudes and latencies were determined and IOFs plotted.

**3. IOFs.** IOFs were plotted for peak amplitude, latency, and also for wave 1–3 inter-peak interval as a function of dB SL. Due to the dependency of amplitude and latency on SPL, it is important to plot IOFs relative to stimulus threshold, so that any changes seen are not a result of variation in response threshold. Parameters for a mutant line were considered significant if the mutant mean value was outside of the reference range for at least 40% of the SLs measured (i.e., for at least 5/12 SLs, when comparing over a 60-dB suprathreshold range).

#### Quality control

All mice issued for phenotyping were genotyped [49] at least twice; once was prior to cohort generation from a clip of pinna skin and a second time was at the end of the pipeline from postmortem skin tissue. Only when both results matched was the genotype for an individual mouse ‘locked’ (confirmed), to allow phenotyping results from that mouse to be included for analyses. ABR results were also subject to a quality control process. Visual inspection of the ABR traces recorded was used to look for significant noise or artefact on the recordings, which was accounted for when allocating threshold and other parameters. For example, excessive myogenic/electrical noise on the recordings can effectively mask an evoked potential, artefactually elevating the threshold estimate. Thresholds were allocated by experienced operators using the criteria outlined above. Data from random mice were checked by a second operator.

#### False positive threshold hits

Our aim was to report robust effects on hearing that are likely to be reproducible in other laboratories, so we were cautious about calling the positive hits. All threshold calls made according to the two criteria detailed above were assessed by two experienced auditory scientists (one of whom was blinded to the genotype) to identify any false positive hits. A small number of false positive hits were discounted based on a number of principles, including excessive variability between individual mice (in some cases due to segregation of an independent new mutation, e.g., [20]) or the presence of a clear outlier (possibly due to a new mutation) in the mutant population skewing the mean threshold. On these grounds the number of false positive calls made by criterion 1 (60% rule) was 6 and by criterion 2 (20dB rule) was 12. Of the 1,211 genes tested, this represents a false discovery rate of 0.50% (criterion 1) and 0.99% (criterion 2).

#### Fisher exact test

We carried out this additional statistical test for information about which threshold calls might be significant if the data had been normally distributed (see above for reasons why this was not our primary test). A two-way contingency table (using the number of threshold observations that were inside or outside the reference range) was generated for each mutant group compared with the wild-type control group, and this was used to carry out a Fisher exact test, giving a *p*-value indicating the likelihood of the two sets of data belonging to the same population (see [7] for details).

### Classification of mouse ABR audiograms

Patterns of raised thresholds for ABRs were classified according to the following criteria: **Severe-Profound**, if no responses were detected (up to 95 dB SPL) for at least two adjacent frequency stimuli, for all mice of that genotype; **High-Frequency**, if thresholds were elevated at 30 kHz (by >30 dB) and thresholds were not elevated for at least one of the lower-frequency stimuli; **Low-Frequency**, if thresholds were elevated for 6 and 12 kHz and were normal for at least one of the higher-frequency stimuli (with a minimum mean threshold elevation <15 dB); **Moderate**, when thresholds were significantly elevated for at least four of the six stimuli tested (with a minimum mean threshold elevation >15 dB); **Mild**, when mean thresholds were elevated by 30 dB or less for up to three stimuli tested; **Normal Hearing**, when no stimuli produced altered thresholds.

### Secondary phenotyping of selected lines

Methods used for histology, immunolabelling and confocal analysis, EP recording, and associated statistical tests used have been published elsewhere [10,11,12,20,42]. ABR thresholds were compared using the Mann-Whitney test.

#### Alleles and genetic backgrounds studied further

The *Srsf7* mutants studied further carried one copy of the mutant *Srsf7*^*tm1a(EUCOMM)Wtsi*^ allele, as homozygotes were subviable, on a C57BL/6N genetic background. *Gpr152* mutants were homozygous for the *Gpr152*^*tm1b(EUCOMM)Wtsi*^ allele on a C57BL/6N background. The *Klc2* mutant allele was *Klc2*^*tm1e(EUCOMM)Wtsi*^ on a C57BL/6N genetic background that had lost the distal LoxP site downstream of its targeted exon (Fig 4K). *Ywhae* mutants studied were homozygous for the *Ywhae*^*tm1e(EUCOMM)Wtsi*^ allele, which had also lost its 3′ LoxP site (Fig 5Q), and were analysed on a mixed C57BL/6N and 129S5/SvEvBrd/Wtsi genetic background because of reduced viability on the original C57BL/6N background.

#### Auditory function recording

ABRs were recorded in new cohorts of *Srsf7*, *Gpr152*, *Klc2*, and *Ywhae* mutants along with littermate controls at 4 weeks, 8 weeks, 14 weeks, and 6 months old, as indicated in Figs 3, 4 and 5, and waveforms were analysed as described previously [10]. EPs were recorded in *Klc2* mutants as described previously [10,11]. The 2f1-f2 distortion product otoacoustic emission (DPOAE) was recorded in *Klc2* mutants in response to f2 frequencies ranging from 6,000 to 30,000 Hz in 500-Hz steps, where the f2:f1 frequency ratio was 1.2 and the f1 and f2 tones were presented at 70 dB SPL and 60 dB SPL, respectively. The 2f1-f2 DPOAE amplitude (dB SPL) was plotted against f2 frequency for control and mutant mice, for comparison.

#### Scanning electron microscopy

Scanning electron microscopy was used to assess the organ of Corti in *Klc2* mutants at 4 weeks old and *Ywhae* mutants at 8–9 weeks old, along with their littermate controls, and the middle ear mucosa of *Ywhae* mutants at 8–9 weeks of age. Inner ears were isolated (7 *Ywhae* homozygotes, 5 *Ywhae* heterozygotes, 2 wild-type littermates; 11 *Klc2* homozygotes, 4 *Klc2* heterozygotes, 2 wild-type littermates) and fixed in 2.5% glutaraldehyde and processed using the osmium tetroxide-thiocarbohydrazide (OTOTO) method as described previously [11]. Middle ear cavities (*n* = 5 *Ywhae* homozygotes, 3 heterozygotes, and 3 wild-type littermates) were prepared by opening the bulla; removing the ossicles, muscles, and ligaments; and fixing in glutaraldehyde and osmium tetroxide, as above. Samples were examined in a Hitachi S-4800 or a JEOL JSM-7610F field emission scanning electron microscope. For the cochlear samples, images of the surface of the organ of Corti were taken at 10% intervals along the length of the cochlear duct or locations corresponding to the best frequency locations of the tones tested in ABR. The frequency areas were determined according to the mouse tonotopic cochlear map described by Müller and colleagues [50].

#### Immunohistochemistry and imaging

Inner ears were fixed in 4% PFA for 2 hours and decalcified in EDTA overnight at room temperature (RT). Following fine dissection, the organ of Corti was permeabilised in 5% Tween PBS for 40 minutes and incubated in blocking solution (4.5 mL of 0.5% Triton X-100 in PBS and 0.5 mL of normal horse serum) for 2 hours. The primary antibodies used overnight at RT were mouse anti-GluR2 (1:200, MAB397, Emd Millipore), rabbit anti-Kcnma1 (1:100, APC-021, Alomone), rabbit anti-Myo7a (1:200, PTS-25-6790-C050, Axxora), mouse anti-Ctbp2 (1:400, BD Transduction Laboratories 612044), and chicken anti-NF-H (1:800, Abcam ab4680). The samples were incubated for 45 minutes at RT with secondary antibodies at 1:500 dilution: goat anti-mouse IgG2a Alexa Fluor 488 (A21131), goat anti-rabbit IgG Alexa Fluor 546 (A11035), donkey anti-mouse Alexa Fluor 594 (Molecular Probes A-21203), and goat anti-chicken Alexa Fluor 488 (Life Technologies A11039) and later were mounted using ProLong Gold mounting media with DAPI and stored at 4°C. Specimens were imaged using a Zeiss Imager 710 confocal microscope interfaced with ZEN 2010 software. The plan-Apochromat 63× Oil DIC objective was used for all the images with 2.0 optical zoom, and confocal z-stacks were obtained with a z-step size of 1 μm for innervation imaging, 0.5 μm for the Myo7a imaging, and 0.25 μm for other images. The frequency areas were determined according to the mouse tonotopic cochlear map described by Müller and colleagues [50].

The number of postsynaptic densities per IHC was quantified by counting manually the GluR2 puncta in the confocal maximum projection images and dividing it by the number of IHC nuclei (DAPI labelled). The cell-counter plugin in Fiji (ImageJ) software was used for counting. Three-dimensional reconstruction of the hair cell confocal stacks was performed using Fiji software (3D project function). Myo7a was labelled with the secondary antibody Alexa Fluor 546 and the nuclei with DAPI; however, false colours were used in the 3D reconstruction.

#### X-gal staining

Heads from mice aged 4 weeks were bisected through the midline, the brain removed, and semicircular canal opened, then fixed in 4% paraformaldehyde at RT for 90 minutes. After decalcification in EDTA, the samples were washed for 30 minutes at RT with rotation with the detergent solution (2 mM MgCl_2_; 0.02% NP-40; 0.01% sodium deoxycholate; 0.01% sodium deoxycholate in PBS, pH 7.3). X-gal (Promega, cat. no. V394A) was added 1:50 to prewarmed staining solution (5 mM K_3_Fe(CN)_6_ and 5 mM K_4_Fe(CN)_6_ in detergent solution), and then the samples were stained at 37°C in the dark overnight. The half-skulls were then washed twice with saline at 4°C in rotation for at least 2 hours and stored at 4°C in 70% ethanol until tissue processing and embedding. The samples were gradually dehydrated in ethanol (Leica TP1020 tissue processor) and embedded in paraffin using xylene as clearing agent (Leica EG1150H tissue embedder). The samples were cut at 8 μm thick, counterstained with Nuclear Fast Red (VWR, cat. no. 342094W), and mounted with Eukitt mounting medium (Sigma-Aldrich). Specimens were imaged using a Zeiss Axioskop microscope connected to an AxioCam camera and interfaced with Axiovision 3.0 software.

#### Skeletal measurements

High-resolution radiographs were collected from 14-week-old *Ywhae* homozygotes (*n* = 6 males, 6 females) with age-, sex-, and strain-matched controls (*n* = 5 males, 6 females) using a Faxitron X-ray cabinet (MX-20, Faxitron X-ray, Wheeling, IL) and assessed using a standard set of parameters, including skull shape, mandible, and teeth [51].

#### Middle ear morphology

External, middle, and inner ear regions were isolated from *Ywhae* mutants aged 16 weeks (4 homozygotes, 3 heterozygotes, 4 wild types). Left sides were examined by dissection for any signs of malformation or inflammation, including excessive cerumen in the external ear canal; thickening, whitening, sponginess, or vascularisation of the bulla wall; clarity of the tympanic membrane; presence of fluid or inflammatory debris in the middle ear cavity; and ossicle malformation. Right ears were fixed for 24–48 hours in formalin, decalcified in EDTA over 2 weeks, embedded in paraffin wax, and sectioned at 5 μm in a coronal plane. Sections were stained with haemotoxylin and eosin, scanned using a Hamamatsu NanoZoomer (Hamamatsu City, Japan), and examined. Examiners of middle ears and sections were blinded to mouse genotype.

#### Western blotting

Brains from 16-week-old mice (2 *Ywhae* homozygotes, 2 heterozygotes, 4 wild types) were snap-frozen in liquid nitrogen for storage at −80°C. Protein extracts were generated by homogenisation in ice-cold T-PER protein extraction reagent (Pierce, Rockford, IL) containing a protease/phosphatase inhibitor cocktail (Halt, ThermoScientific, Waltham, MA). Protein (50 μg) from each sample was run in 4%–12% Bis-Tris gels (Life Technologies, Paisley, UK). A primary rabbit polyclonal antibody directed against a peptide mapping within a divergent domain of human YWHAE was used to detect the protein (T-16, sc1020, Lot: C1914; 1:200, Santa Cruz Biotechnology, Dallas, TX; Antibody Registry AB_630821) [52], PAFAH1b1/LIS1 was detected with recombinant rabbit monoclonal raised against a synthetic peptide corresponding to residues in human LISs1 (ab109630, Lot: GR55503-8; 1:750, Abcam, Cambridge, UK; Antibody Registry AB_10861275), and a blot with an additional LIS1/PAFAH1b1 rabbit polyclonal antibody generated against synthetic peptide corresponding to residues surrounding Gly298 of human LIS1 protein was used for validation (12453S Lot:1, Cell Signaling Technology, Danvers, MA). Goat anti-rabbit monoclonal vinculin antibody was used as a loading control (ab129002, 1:10,000, Abcam; Antibody Registry AB_11144129). A goat anti-rabbit IgG HRP-conjugate was used as a secondary antibody (12–348, Lot 2584427; Millipore, Billerica, MA; Antibody Registry AB_390191). The blots were imaged by enhanced chemiluminescence using an ImageLAS 4000 system (GE Healthcare, Chalfont St. Giles, UK).

### Bioinformatic analysis of the 1,211 genes screened

GO term enrichment was analysed using FuncAssociate v3.0 ([53] http://llama.mshri.on.ca/funcassociate/), based on 22,644 genes (genespace), with 19,064 GO attributes, downloaded on 22 December 2015. FuncAssociate was configured to exclude computationally predicted GO annotations (Inferred from Electronic Annotation [IEA] evidence code) and run against the 1,211 genes tested by ABR. Revigo was used to reduce the redundancy in GO terms classed as over- or under-represented (by FuncAssociate) by clustering significant terms into more representative subsets ([54] http://revigo.irb.hr/). Only 0.42% GO terms (*n* = 80) out of 19,064 were overrepresented in our list of 1,211 genes, and 0.09% (*n* = 18) were under-represented. The over- and under-represented GO attributes were not significantly clustered into distinct subsets (visualised as TreeMaps). We also analysed this list of genes with the Reactome pathway database using Reactome V58 (www.Reactome.org), released October 2016; 10,168 human reactions were organised in 2,069 pathways involving 10,212 proteins and 10,214 complexes. Of the 1,211 genes tested, 564 were not listed in Reactome (46.6%). Of the 38 new genes underlying increased ABR thresholds, only 12 were included in Reactome. Of the 27 genes associated with abnormal ABR waveforms, only 17 were included in Reactome. Of 362 deafness genes already known, 233 were found in the Reactome databases. The 647 (53.4%) genes represented in the Reactome database out of the initial 1,211 did not produce any overrepresentation of any particular pathways (false discovery rate probability, FDR > 0.839).

Thus, taking the GO term and Reactome analyses together, the 1,211 mouse genes targeted can be considered to be representative of the entire mouse genome.

### GO analysis of genes associated with hearing abnormalities

We used the GOSlim analysis tool on the MGI website (http://www.informatics.jax.org/gotools/MGI_GO_Slim_Chart.html) to compare the proportions of high-level GO terms associated with genes in each of five lists: new genes associated with raised ABR thresholds (*n* = 38); previously known genes associated with raised thresholds, human and mouse (*n* = 362); new genes associated with ABR waveform abnormalities (*n* = 27); all genes screened by ABR (*n* = 1,211); and all genes in MGI (*n* = 33,395). We excluded evidence code IEA. The proportions of genes labelled with each high-level GO term in each group are plotted in Fig 2.

### Variant identification in candidate genes in human deafness

The hearing-impaired child was ascertained through the Kaiser Permanente clinic, testing was performed at Ambry Genetics, and the mutations were identified in *SPNS2* by sequencing of candidate genes as part of clinical whole exome analysis. Variant pathogenicity prediction was carried out as previously described [55,56]. The link to the mouse study was established through GeneMatcher [57].

### Human population analysis

The 1958 British Birth Cohort and the collection of hearing data and analysis have been described previously [58,59,60]. Participants were drawn from 17,638 individuals born in England, Scotland, and Wales in one week of March 1958. Of the original cohort, 9,377 members were revisited by a research nurse for a biomedical follow-up in 2002–2004. Hearing measures consisted of pure tone audiometry at 1 kHz and 4 kHz at age 44–45 years and were adjusted for sex, nuisance variables (noise at test, nurse performing test, audiometer used in test), conductive loss, and hearing loss in childhood. DNA was collected from 6,099 individuals and genotyped on various Illumina and Affymetrix SNP chips (for detail, see http://www2.le.ac.uk/projects/birthcohort/1958bc/available-resources/genetic). These data were then imputed to the 1,000 Genomes haplotypes (released March 2012) using MACH and Minimac. Measured SNPs with >95% call rate and Hardy–Weinberg *p*-value >0.0001 were included as the input set. In subsequent analysis, imputed SNPs with low imputation quality (r2-hat < 0.3 or MAF < 1%) were omitted. For the association analysis, hearing thresholds at 1 kHz and 4 kHz were log transformed and adjusted for sex, nuisance variables, and conductive hearing loss in childhood; they were analysed by performing a 1-df ‘per allele’ significance test for association between mean hearing threshold and number of minor alleles (0, 1, or 2) as described previously [60]. The Bonferroni-corrected *p*-value for this candidate gene analysis is *p* < 6.76 × 10^−4^, based on testing 37 genes against two threshold frequencies (1 kHz and 4 kHz) at a significance *p*-value of 0.05, so all *p*-values listed are significant after correction for multiple testing. A *p*-value of *p* < 6.76 × 10^−4^ represents the top 0.06% and 0.08% associations of all approximately 9 million imputed variants for 1 kHz and 4 kHz, respectively.

## Supporting information

S1 Fig ABRs, threshold estimation, and click-evoked waveform shape and parameters. ABRs evoked by clicks (A), 6-kHz tones (B), 12-kHz tones (C), 18-kHz tones (D), 24-kHz tones (E), and 30-kHz tones (F) in a normal hearing wild-type mouse (black lines; 0–85 dB SPL) and a *Duoxa2* homozygous mutant (red lines; 0–95 dB SPL). Threshold estimates are indicated by thickened black and red lines. In the case of the *Duoxa2* mutant, no responses were measurable up to 95 dB SPL, and this sound level was arbitrarily assigned to be threshold. G. Threshold estimates from the responses from the two mice illustrated in A–F are plotted for the normal hearing wild-type mouse (black) and the *Duoxa2* mutant mouse (red). The green area plotted indicates the 2.5–97.5 percentile range of thresholds for each stimulus, recorded from mice on the C57BL/6N genetic background, and represents the 95% reference range used to assess whether mutant mice have normal or elevated thresholds. H. ABR waveforms are illustrated for click stimuli at 50 dB above threshold in the normal hearing mouse (black line) and the 95 dB SPL click stimulus in the *Duoxa2* mutant mouse (red line). The four waves of the click-evoked ABR are indicated by the grey areas, and the four positive and four negative peaks are labelled P1–P4 and N1–N4. Panels I-M indicate IOFs derived from features of the click-evoked responses recorded over a 60-dB range of stimulus levels above threshold. As above, the green area plots the 2.5–97.5 percentile range of each parameter and represents the 95% reference range used to assess whether mutant mice have normal or abnormal IOFs. I. IOF for wave 1 amplitude (P1-N1 amplitude). J. IOF for wave 3 amplitude (P3-N3 amplitude). K. IOF for latency of P1. L. IOF for latency of P3. M. IOF for the interval between P1 and P3. Plotted data points are given in S1 Data. ABR, auditory brainstem response; IOF, input-output function.
(TIF)

S2 Fig ABR thresholds of positive controls, new alleles of genes known to underlie hearing impairment, and male and female wild-type mice. ABR thresholds (dB SPL) are plotted against stimulus frequency and clicks. On each panel, green triangles and lines represent the mean thresholds (±SD) for control mice recorded in the same week as the mutants, and red circles and lines represent the mean thresholds (±SD) for mutant mice. Thresholds for individual mutants are shown by open grey circles and lines. Green bands denote the 95% reference range for a large population of control wild-type mice. Top panel: (A-J) Pre-existing mutant mouse lines known to have hearing impairment, tested as positive controls to validate the methodology used in this screen. Mutant lines tested were (A) *Chd7*^*Whi*^, wild-type versus heterozygous mutants; (B) *Atp2b2*^*Obv*^, wild-type versus heterozygous mutants; (C) *Myo7a*^*Hdb*^, wild-type versus homozygous mutants; (D) *Myo7a*^*sh1-6J*^, heterozygous controls versus homozygous mutants; (E) *Cdh23*^*v*^, heterozygous controls versus homozygous mutants; (F) *Grxcr1*^*Tde*^, heterozygous controls versus homozygous mutants; (G) *Mir96*^*Dmdo*^, wild-type versus homozygous mutants; (H) *Myo6*^*sv*^, heterozygous controls versus homozygous mutants; (I) *Tmc1*^*dn*^, heterozygous controls versus homozygous mutants; and (J) *Whrn*^*wi*^, heterozygous controls versus homozygous mutants. Middle panel: (K-T) New targeted alleles of known deafness genes: (K) *Myo15*^*tm1a(EUCOMM)Wtsi*^, (L) *Myo7a*^*tm1a(EUCOMM)Wtsi*^, (M) *Ush1c*^*tm1a(KOMP)Wtsi*^, (N) *Ildr1*^*tm1(KOMP)Wtsi*^, (O) *Espn*^*tm1a(EUCOMM)Wtsi*^, (P) *Whrn*^*tm1a(EUCOMM)Wtsi*^, (Q) *Cep250*^*tm1a(EUCOMM)Wtsi*^, (R) *Srrm4*^*tm1e(EUCOMM)Wtsi*^, (S) *Clpp*^*tm1a(EUCOMM)Wtsi*^, and (T) *Chd7*^*tm2a(EUCOMM)Wtsi*^ (included as an example of normal thresholds). Bottom panel: (U-X) Mean (±SD) threshold for male (red) and female (black) mice are plotted for (U) Mouse GP pipeline, mixed C57BL/6Brd-*Tyr*^*c-Brd*^;C57BL/6N line (B6JTyr/B6N) (female *n* = 243, male *n* = 237), (V) Mouse GP pipeline, C57BL/6N line (B6N) (female *n* = 191, male *n* = 180), (W) MGP Select pipeline, mixed B6JTyr/B6N (female *n* = 29, male *n* = 25), (X) MGP Select pipeline, C57BL/6N (female *n* = 120, male *n* = 117). Plotted data points are given in S2 Data. ABR, auditory brainstem response; MGP, Mouse Genetics Project; SPL, sound pressure level.
(TIF)

S3 Fig Genes affecting central auditory system function with normal thresholds. Mutant mouse lines showing altered ABR waveforms but normal thresholds are shown. ABR thresholds are plotted on the left of each set of three plots. Averaged click-evoked ABR waveforms recorded at 50 dB above threshold are plotted in the middle column of each set. IOFs for selected abnormal features are plotted on the right of each set. The green band denotes the 95% reference range of control values. Red lines and circles represent mean responses (±SD) from mutant mice. Responses from individual mutants are shown by grey lines and open grey circles. Green lines and circles represent the mean thresholds (±SD) for control mice recorded in the same week as the mutants. Plotted data points are given in S3 Data. ABR, auditory brainstem response; IOF, input-output function.
(TIF)

S4 Fig Genes affecting central auditory system function and thresholds. Mutant mouse lines showing raised thresholds and altered ABR waveforms are shown. ABR thresholds are plotted on the left of each set of three plots. Averaged click-evoked ABR waveforms recorded at 50 dB (unless stated otherwise on the panel) above threshold are plotted in the middle column of each set. IOFs for selected abnormal features are plotted on the right of each set. The green band denotes the 95% reference range of control values. Red lines and circles represent mean responses (±SD) from mutant mice. Responses from individual mutants are shown by grey lines and open grey circles. Green lines and circles represent the mean thresholds (±SD) for control mice recorded in the same week as the mutants. Plotted data points are given in S4 Data. ABR, auditory brainstem response; IOF, input-output function.
(TIF)

S1 Table A summary of features of interest identified from ABR measurements in mutant mice. On the ‘Gene List’ worksheet, columns (left to right) denote gene symbol, allele tested, genotypes compared, genetic background strain, and number of mutant mice tested. The heat map represents click-evoked, 6-kHz, 12-kHz, 18-kHz, 24-kHz, and 30-kHz ABR thresholds, click-evoked ABR waveform overlay of all mice tested at the same stimulus level above threshold, and IOFs of features of the positive (P) and negative (N) peaks of the ABR waveform, including P1-N1, N2-P3, P3-N3 amplitude; P1, N1, P3, and N3 latency; and P1-P3 and N1-N3 interval. Blue cells indicate a parameter within the normal range. Red cells indicate abnormal parameters (as detailed in Methods). For threshold estimates, a red cell containing ‘20dB’ was called on the basis of the mutant mean deviating by ≥20dB from the reference range median value; all other red cells were called on the basis that 60% of observations or more fell outside the reference range. For click-evoked ABR waveform shapes, a red cell indicates that the observation of the overlaid waveforms suggested an abnormal response and that this was supported by subsequent IOF quantification; an orange cell indicates that overlaid waveforms suggested an abnormal response but this was not supported by input-output analyses. For input-output analyses, a red cell indicates the parameter was outside the reference range for at least 40% of the sound levels tested, and the arrow indicates the direction of change. A cell containing a dash (-) indicates that input-output analysis was not carried out, as waveforms appeared normal. Grey cells (containing ‘n/a’) indicate that data were not available for analysis; for example, thresholds were too high to allow waveform overlays or that waveforms were too abnormal to permit IOF analysis. Genetic background: B6N, C57BL/6N; B6JTyr;B6N, mixed C57BL/6Brd-*Tyr*^*c-Brd*^ and C57BL/6N; C3Fe, C3HeB/FeJ; B6JIco;B10, mixed C57BL/6JIco and C57BL/10ScSn; AK;BKS, AKR/J 25.0% and C57BLKS/J 75.0%; STOCK, partly undefined background. Further columns indicate mouse gene symbol, mouse Ensembl ID, human Ensembl ID, and gene symbol of the human orthologue. Other columns summarise the other phenotyping information obtained from the Mouse Genetics Project screen at the Wellcome Trust Sanger Institute. White cells indicate that data are not available; blue cells indicate no significant abnormalities; red cells indicate that one or more parameters varied significantly from control values. This heat map can be examined in more detail by clicking on the links on the top row, which links to other worksheets showing the individual parameters tested in each test area. In these worksheets, only parameters considered to vary significantly from control values are shown, plotted as red cells. Where appropriate, the direction of the change of the parameter is indicated by an up or down arrow. Cells containing ‘M’ or ‘F’ indicate that the change was significant for male or female mice only. White cells indicate that either the data were not collected or the results were not significant. On these worksheets, the link in cell E1 (‘Back to Gene List’) returns the user back to cell A1 of the main Gene List worksheet. ABR, auditory brainstem response; ID, identifier; IOF, input-output function.
(XLSX)

S2 Table A summary of ABR results from all mutant lines tested. See S1 Table legend for details of columns and key to data representation. Additional columns to the right indicate the significant calls for each stimulus threshold, with red where *p*-value is greater than the appropriate critical value and blue where the *p*-value is not significant, using a two-way contingency table and the Fisher exact text. For new mutant lines tested, the Bonferroni-corrected critical *p*-value = 4.1 × 10^−5^, and for positive control lines, the critical *p*-value = 0.05, as these were compared with littermate controls due to their unique genetic background. Mutant lines tested are grouped in rows in the following categories: (1) Mutant mouse lines screened when gene was previously reported to be associated with hearing impairment. (2) Mutant mouse lines of known deafness genes where the mouse line did not show raised ABR thresholds. (3) Comparison of mutant lines when more than one allele was screened. For both *Rnf10* and *Selk*, the *tm1a* allele responses were normal, but the *tm1b* allele responses showed an abnormal feature. For a further two genes, *Spns2* and *Zfp719*, responses from mice carrying the *tm1b* allele were marginally more affected than those from mice carrying the *tm1a* allele. In one unusual case, a targeted mutation in the *Slc25a21* gene only produced affected ABR responses for the *tm1a* allele, where the inserted cassette of DNA was found to interrupt expression of the neighbouring *Pax9* gene and result in phenotype differences [8]. Once this cassette was removed to produce the ‘b, ‘c’, and d’ alleles, expression of *Pax9* was restored, and the normal ABR phenotypes were also restored. (4) The main grouping of the table lists a summary of the ABR results for all the mutant lines tested, listed alphabetically. ABR, auditory brainstem response.
(XLSX)

S3 Table Known deafness genes with normal ABR thresholds in the MGP Screen. This table lists the genes that were previously known to be involved in deafness either in mouse or human, but which did not show raised ABR thresholds in the current study. The genotype reported deaf and the genotype screened are indicated. The right-hand column indicates possible explanations for the discrepancy in each case. ABR, auditory brainstem response; DFNA, non-syndromic deafness with dominant inheritance; DFNB, non-syndromic deafness with recessive inheritance; DFNX, non-syndromic deafness with X-linked inheritance; Hemi, hemizygote; Het, heterozygote; Hom, homozygote; MGP, Mouse Genetics Project; tm, targeted mutation.
(DOCX)

S4 Table Features of genes identified in this ABR screen. This table summarises information from a range of sources to indicate features of interest about the genes detected with ABR defects in this screen, illustrating the broad range of types of genes found associated with hearing impairment. Columns A–I and K–P were derived from the MGI database, with links given in column E. For column M, references to phenotype details of alleles reported here are not included, only reports of other alleles of the same gene. For column P, only selected key GO terms are listed. Column J was obtained by comparing the human gene location with unidentified non-syndromic hearing loss loci listed in the Hereditary Hearing Loss Homepage (Van Camp and Smith; http://hereditaryhearingloss.org/; accessed January 2019). Column Q was derived from violin plots of single-cell mRNAseq data presented in the gEAR portal (Herzano and colleagues; https://umgear.org; accessed January 2019). The criteria for inclusion of the genes were either (1) threshold above 95% confidence interval for at least one stimulus in 60% of mice of that genotype tested in the MGP screen, (2) *mean threshold for at least one stimulus more than 20 dB above wild-type mean, or (3) †further data obtained after screen confirmed raised thresholds in a larger *n*. All hits listed were in homozygotes unless noted as *(Het)* in column A when heterozygotes were screened. Four or more mice were screened unless a lower number is given in column A. ABR, auditory brainstem response; GO, Gene Ontology; MGI, Mouse Genome Informatics; MGP, Mouse Genetics Project; mRNAseq, messenger RNA sequence.
(XLSX)

S1 Data Data for S1 Fig. (XLSX)

S2 Data Data for S2 Fig. (XLSX)

S3 Data Data for S3 Fig. (XLSX)

S4 Data Data for S4 Fig. (XLSX)

S5 Data Data for Fig 1. (XLSX)

S6 Data Data for Fig 2. (XLSX)

S7 Data Data for Fig 3. (XLSX)

S8 Data Data for Fig 4. (XLSX)

S9 Data Data for Fig 5. (XLSX)

S10 Data Data for Fig 6. (XLSX)

## Abbreviations

ABR: auditory brainstem response
DPOAE: distortion product otoacoustic emission
EP: endocochlear potential
ER: endoplasmic reticulum
ES: embryonic stem
f: frequency
FRT: flippase recombinase target
GO: Gene Ontology
IEA: Inferred from Electronic Annotation
IHC: inner hair cell
IOF: input-output function
LacZ: gene encoding β-galactosidase
LoxP: locus of crossover in P1 bacteriophage
MGI: Mouse Genome Informatics
MGP: Mouse Genetics Project
OHC: outer hair cell
OTOTO: osmium tetroxide-thiocarbohydrazide
P: postnatal day
qRT-PCR: quantitative real-time PCR
RT: room temperature
SL: sensation level
SPL: sound pressure level
S1P: sphingosine-1-phosphate
tm: targeted mutation
