# Supplementary material for: Mouse screen reveals multiple new genes underlying mouse and human hearing loss
Source: PLoS Biol. 2019 Apr 11;17(4):e3000194. doi: 10.1371/journal.pbio.3000194 (PMC6459510; doi:10.1371/journal.pbio.3000194)
Supplement: S3 Table — This table lists the genes that were previously known to be involved in deafness either in mouse or human, but which did not show raised ABR thresholds in the current study. The genotype reported deaf and the genotype screened are indicated. The right-hand column indicates possible explanations for the discrepancy in each case. ABR, auditory brainstem response; DFNA, non-syndromic deafness with dominant inheritance; DFNB, non-syndromic deafness with recessive inheritance; DFNX, non-syndromic deafness with X-linked inheritance; Hemi, hemizygote; Het, heterozygote; Hom, homozygote; MGP, Mouse Genetics Project; tm, targeted mutation. (DOCX) [file pbio.3000194.s007.docx]

**S3 Table. Known Deafness Genes with normal ABR thresholds in the MGP Screen.**

| **Gene** | **Phenotype** | **Species reported deaf** | **Genotype reported deaf** | **Genotype and allele screened in MGP** | **Possible explanation for no ABR phenotype** |
| --- | --- | --- | --- | --- | --- |
| *GSDME*  *(DFNA5)* | DFNA5 | Human | Het, skipping of exon 8, possible dominant negative | Hom, *tm1b* | Specific dominant negative mutation |
| *MYH14* | DFNA4 | Human | Het, missense, nonsense | Hom, *tm1a* | Specific effect of missense mutation or leaky *tm1a* allele |
| *MYH9* | DFNA17 | Human | Het, missense | Het, *tm1a* | Specific effect of missense mutation or leaky *tm1a* allele |
| *PNPT1* | DFNB70 | Human | Hom, missense | Het, *tm1a* | Homozygote not screened |
| *PRPS1* | DFNX1 | Human | X-linked, missense | Het, *tm1a* | Hemizygote/homozygote not screened |
| *CHD7* | CHARGE syndrome | Human | Het, often *de novo* | Het, *tm2a* on mixed genetic background | Incomplete penetrance of hearing impairment in human; genetic background has impact on het viability so maybe also on hearing |
| *Barhl1* | Progressive hearing loss | Mouse | Hom, null | Het, EGFP_CreERT2 | Homozygote not screened |
| *Fzd6* | Hair bundle polarity defect | Mouse | Double Hom *Fzd3* and *Fzd6* nulls | Hom, *tm2a* | Redundancy with *Fzd3* |
| *Hmx3* | Raised thresholds | Mouse | Hom, null | Hom, *tm1Ebo* | Same allele tested, previous report used compound action potentials and mutants showed variable defects |
| *Nfkb1* | Progressive hearing loss | Mouse | Hom null | Hom, *tm1a* | Slow rate of progression not detected at 14 weeks or leaky *tm1a* allele |
| *Sgms1* | Progressive hearing loss | Mouse | Hom, null | Hom, *tm1a* | Slow rate of progression not detected at 14 weeks or leaky *tm1a* allele |
| *Sms* | Deafness and balance defect | Mouse | Deletion of 4 genes | Hemi male, Het female, *tm1a* | Other deleted genes in the *Gyro* mutant may contribute |
| *Synj2* | Progressive hearing loss | Mouse | Hom, missense | Hom, *tm1a* | *tm1a* allele is leaky |
